# Recent Progress in Mechanoluminescence for Multi‐Dimensional Stress Monitoring

**DOI:** 10.1002/advs.202519938

**Published:** 2025-12-08

**Authors:** Xiuxia Yang, Ting Wang, Lei Zhao, Xuhui Xu

**Affiliations:** ^1^ Faculty of Materials Science and Engineering Key Laboratory of Advanced Materials of Yunnan Province Kunming University of Science and Technology Kunming Yunnan 650093 P. R. China; ^2^ College of Materials and Chemistry & Chemical Engineering Nuclear Technology Key Laboratory of Earth Science Chengdu University of Technology Chengdu 610059 P. R. China; ^3^ School of Physics and Opto‐Electronic Technology Collaborative Innovation Center of Rare‐Earth Optical Functional Materials and Devices Development Baoji University of Arts and Sciences Baoji Shaanxi 721016 P. R. China

**Keywords:** 0D point detection, 1D linear distribution, 2D planar imaging, 3D volume reconstruction, mechanoluminescence

## Abstract

Mechanoluminescence (ML) materials with their unique stress‐to‐light conversion capability, have been driving the evolution of stress sensing technology from single‐point detection to multi‐dimensional imaging. Recent advances in material defect engineering, ion doping, and heterostructure design have significantly improved the luminosity, response kinetics, and environmental stability of ML materials. These enhancements have established a technical foundation for multimodal stress visualization across diverse application scenarios. To systematically analyse the stress response mechanism and visual behavior of materials across different spatial dimensions, this review offers in‐depth discussions on material design principles, device integration methods, and application scenarios for 0D, 1D, 2D, and 3D stress sensing technologies. It further proposes targeted solutions to common challenges in multi‐dimensional stress sensing, such as material performance, device integration synergy, and data processing. Finally, the development path of multi‐dimensional stress visualization technology will be looked forward with higher spatial resolution in intelligent diagnosis, biomechanics, and extreme environmental monitoring, providing a theoretical framework and technical support for multi‐dimensional stress visualization detection.

## Graphical abstract

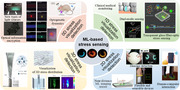

## 1 Introduction

ML materials are a new type of optoelectronic functional materials that can produce light under the stimulation of mechanical stress (the mechanical stress can be applied through fracture, friction, extrusion, impact, etc.).^[^ ^1^, ^2^, ^3^, ^4^, ^5^, ^6^, ^7^, ^8^ ^]^ In 1605, British scholar Francis Bacon discovered that a faint glow occurred when cutting sugar cubes with a blade, and recorded the phenomenon in the book of “The Advancement of Learning”. Subsequently, ML has been identified in an increasing number of natural minerals and compounds. For example, in 1644, the British Robert Boyle observed that natural diamond emitted transient light upon mechanical stress.^[^ ^9^, ^10^, ^11^, ^12^ ^]^ Due to the limitations of scientific research conditions at that time, the phenomenon has been consequently documented simply as a novel form of luminescence. Since the inception of systematic research in the 1990s, ML materials have exhibited distinctive advantages for stress‐sensing applications.^[^ ^13^, ^14^, ^15^ ^]^ Early ML research primarily focused on single‐point stress detection, exploiting the linear relationship between localized luminous intensity and mechanical stress to enable threshold sensing and microstrain response.^[^ ^16^, ^17^, ^18^, ^19^ ^]^ However, the growing need for complex stress field visualization in applications such as structural health monitoring, biomechanical analysis, and human‐machine interaction is driving the from point‐based stress detection toward spatially resolved 3D field mapping. Multi‐dimensional stress visualization technology (0D point detection, 1D linear distribution, 2D planar imaging, 3D volume reconstruction) has become a focus of attention in the field of stress sensing. The transition from “points” to “multi‐dimensional spatial fields” facilitates real‐time, in situ, and high‐resolution visualization of stress distribution, thereby enabling applications in structural damage warning, biological tissue mechanics behavior analysis, and human‐computer interaction interfaces.^[^ ^20^, ^21^, ^22^, ^23^, ^24^ ^]^

At the fundamental research level, advances in the multi‐dimensional ML field mapping primarily stem from three critical developments: First, optimization of material optical properties through bandgap engineering and defect structure control has substantially enhanced stress‐to‐light conversion efficiency.^[^ ^25^, ^26^, ^27^, ^28^, ^29^ ^]^ For example, in 2007, Xu et al. co‐doped Ho^3+^ ions into SrAl_2_O_4_:Ce material to introduce shallow traps, thereby significantly improving the material's ML performance.^[^ ^30^ ^]^ In 2019, Wang et al. developed a ZnS/CaZnOS heterostructure that used interfacial bonding‐induced band offsets to reduce the energy barrier for electron excitation into the conduction band, thereby enhancing radiative recombination efficiency and achieving highly efficient, stable, and reproducible ML.^[^ ^31^ ^]^ This laid the foundation for high‐sensitivity stress sensing. Second, from the perspective of device composition design, researchers have notably enhanced the performance and environmental adaptability of the ML sensing system through optimization of the layered structure.^[^ ^32^, ^33^, ^34^, ^35^ ^]^ For example, in 2015, Wang et al. designed an electronic signature sensing system using a layered structure. By converting stress information into synchronously collectable optical signals, they achieved significant improvements in stress imaging response time.^[^ ^36^ ^]^ This approach enabled the construction of a handwritten trajectory recognition system and established a design framework for multi‐dimensional stress‐sensing networks. Finally, regarding particle size regulation in material synthesis, Hong et al. fabricated ZnS:Ag,Co@ZnS core@shell nanoparticles via a hydrothermal synthesis technique.^[^ ^37^ ^]^ These particles can be charged by 400 nm light (energy stored in electronic traps) as they flow through superficial blood vessels. Subsequent focused ultrasound (FUS) stimulation triggers localized 470 nm blue emission in deep brain regions. The photonic energy release can activate light sensitive ion channels (channelrhodopsin‐2, ChR2), which opens up the possibility for the development of bio‐ultrasound imaging and non‐invasive optogenetic techniques.

**Figure 1:**
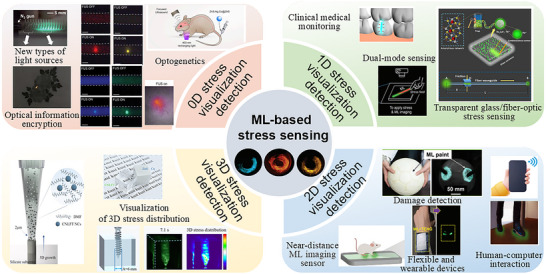
The overview of multi‐dimensional application scenarios of ML materials.

From an application perspective, the 0D single‐point ML sensing system relies on local ML intensity to achieve qualitative stress visualization detection, enabling applications in new light sources,^[^ ^38^, ^39^ ^]^ optical information encryption,^[^ ^40^, ^41^ ^]^ bio‐ultrasound imaging,^[^ ^42^, ^43^ ^]^ and ultrasound‐controlled optogenetic technology.^[^ ^44^, ^45^ ^]^ The 1D linear ML sensing system utilizes distance and ML intensity for visual sensing, supporting applications such as distributed stress mapping or real‐time trajectory monitoring.^[^ ^46^, ^47^ ^]^ The 2D ML sensing system leverages biaxial planar position and ML intensity to achieve visual detection of planar stress distribution, facilitating damage detection in complex components during operation,^[^ ^48^ ^]^ flexible wearables,^[^ ^49^, ^50^ ^]^ and human‐machine interaction interfaces.^[^ ^51^, ^52^ ^]^ The 3D ML sensing system depends on the ML intensity and volumetric position information to achieve visualization and quantitative analysis of the internal stress/strain distribution of objects. It can be used for visualization and detection of stress distribution within 3D structures.^[^ ^21^, ^22^ ^]^ In summary, this review will systematically analyse the research progress of ML materials from 0D to 3D from the perspective of multi‐dimensional stress sensing applications of ML materials (**Figure** **1**). First, we will discuss the principles of material design and performance control strategies from different dimensions. Then, we will focus on summarising the opportunities and challenges of multi‐dimensional ML sensing technology in fields such as intelligent sensing, biomedicine, and flexible electronics. Finally, we will look ahead to future trends, particularly the innovative prospects of 4D (3D spatial + time) dynamic sensing systems with real‐time feedback capabilities.

## 2 0D Stress Visualization Detection

The 0D stress visualization detection technology exploits the localized stress response characteristics of ML materials. Through dynamic analysis of single‐point fluorescence signals at material sites (such as microscopic defects, interfaces, or stress concentration zones), it enables precise visualization and characterization of microscopic mechanical behaviors. At present, two main technical paths have been developed: 1) the design and synthetic of ML materials with high stress sensitivity, where the optical property exhibit significant stress‐dependent modulations at localized regions. 2) the development of a miniaturized implantable and adhesive ML sensor enabling high‐sensitivity point stress visualization detection. This 0D stress visualization technology demonstrates considerable promise across interdisciplinary domains.

### 2.1 New Light Sources

**Figure 2:**
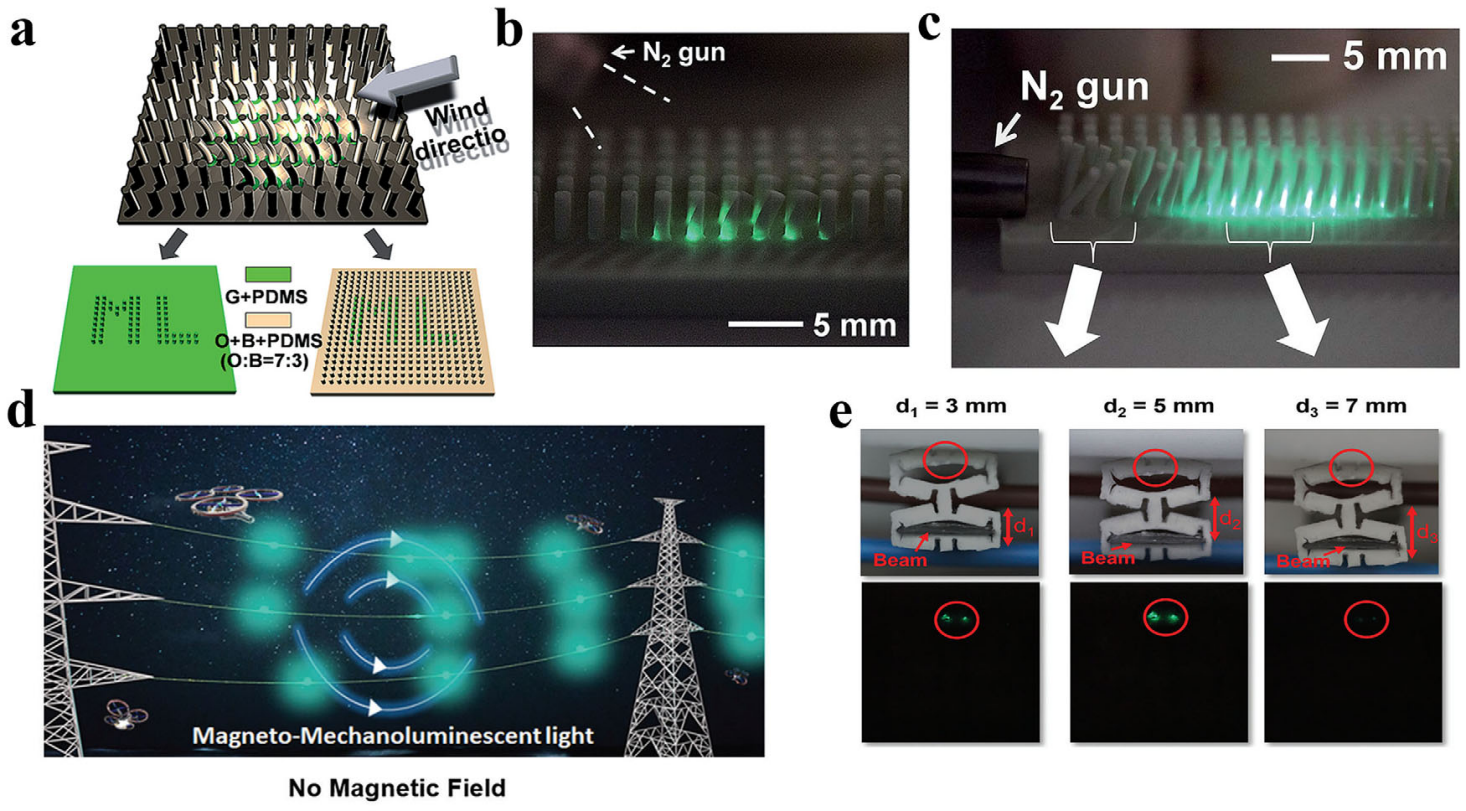
a) Schematic illustration of the set‐up; b) Photograph of the ML image showing the distribution of the ML emission in the vibrating rods; c) Photograph of the wind‐driven ML image under continuous gas flow, Reproduced with permission.^[^ ^38^ ^]^ Copyright 2014, Wiley‐VCH; d) Illustration of the obstacle warning light systems when attached to transmission power lines for improving visibility and reducing the risk of flying object strikes; e) The photos show the luminescence exerted under practical conditions with variations in the gap dimensions between cables, Reproduced with permission.^[^ ^39^ ^]^ Copyright 2023, Wiley‐VCH.

0D stress visualization detection technology utilizes the localized stress‐responsive characteristics of ML materials to achieve stress‐dependent emission. Through modulation of emission wavelength and intensity by stress, dynamically tunable light sources can be obtained. At present, the display devices based on ML material have been successfully developed, which can drive by wind, electric field‐induced mechanical stress, and magnetic force. In 2014, Jeong et al. fabricated a flexible rod array structure by compositing the highly stress‐sensitive ML material ZnS:Cu with polydimethylsiloxane (PDMS) elastomer to achieve 0D localized stress visualization and detection^[^ ^38^ ^]^ As illustrated in **Figure** **2**a–c, the nitrogen gas has been used to simulate natural wind, driving the deformation of the flexible rod array and thereby inducing localized high‐brightness ML for stress visualization. In 2023, Song et al. developed a magneto‐ML (MML) device composed of ZnS:Cu and PDMS.^[^ ^39^ ^]^ This material can be integrated with a magneto‐mechano‐vibration (MMV) cantilever beam to achieve a power‐free, magnetically driven lighting device. Through finite element analysis (FEA) and optical characterization, the shape and dimensions of the Kirigami‐shaped ML composite are optimized to achieve optimal stress distribution and emission intensity. As shown in Figure 2d,e, the ML composite is capable of generating locally visible light under a 60 Hz alternating magnetic field, enabling efficient emission without complex electrical energy conversion steps. Although the current device exhibits relatively weak emission intensity, its performance in practical environments demonstrates promising potential as a power‐free lighting system, offering new perspectives for the future development of sustainable illumination technologies.

### 2.2 Optical Information Encryption

**Figure 3:**
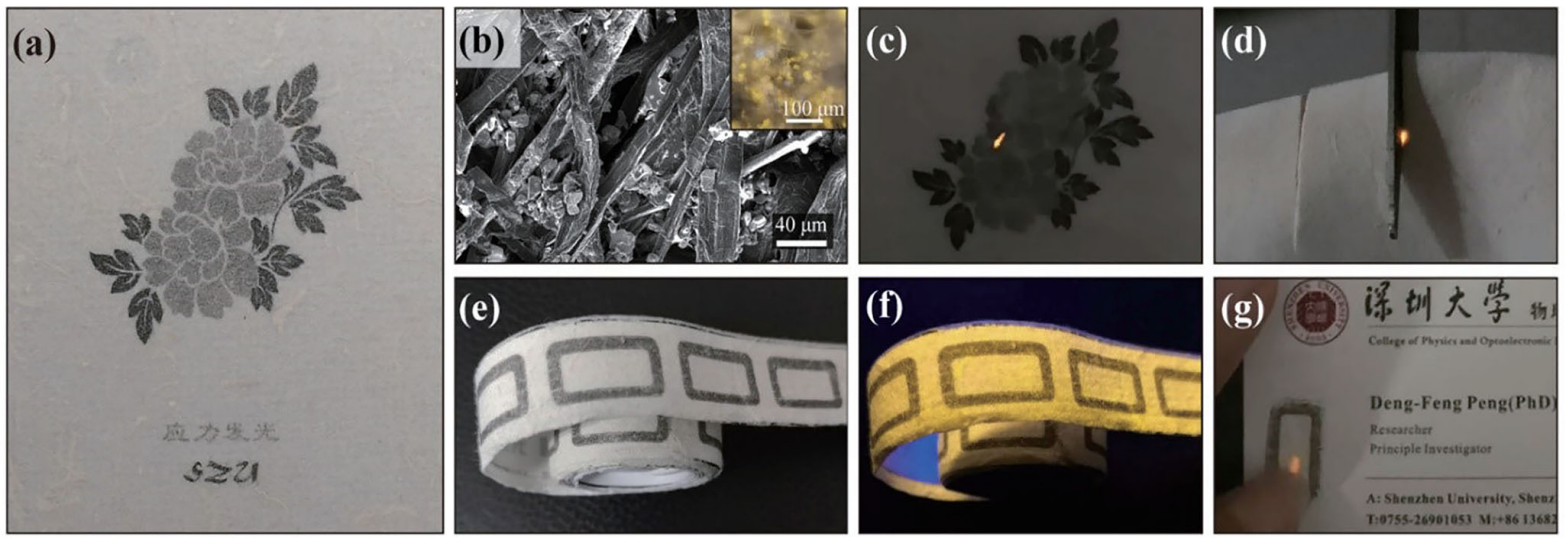
a) The photo of ML paper made in the laboratory with directly inkjet‐printed patterns; b) SEM and (inset) fluorescent microscope images of a dried paper‐making mixture of ML phosphor and wood pulp; ML recorded during c) scratching on the back of the printed pattern and d) cutting the paper by scissors; ML label illuminated by e) daylight and f) 365 nm UV lamp and g) its usage in anti‐counterfeiting, Reproduced with permission.^[^ ^56^ ^]^ Copyright 2022, Springer Nature.

The advancement of modern science and technology has brought convenience to people's lives, but it has also made counterfeiting an effortless job for fraudsters. The proliferation of counterfeit products has disrupted the normal economic order, caused significant financial losses, and exerted negative impacts on human life. 0D stress visualization detection technology, utilizing the point‐specific and unreplicable stress‐fluorescence response of ML materials, has enabled the development of stress‐activated dynamic anti‐counterfeiting encoding techniques, offering a higher level of coding technology for advanced anti‐counterfeiting applications.^[^ ^53^, ^54^, ^55^ ^]^ In 2021, Peng et al. synthesized ZnS:Mn^2+^ via salt‐shielded method. By precisely regulating the phase ratio and achieving large‐scale production, they fabricated sphalerite/wurtzite heterojunction‐structured ZnS:Mn^2+^.^[^ ^56^ ^]^ Combined with DFT calculations, they analyzed and confirmed that defects activate local S and Zn ion sites and reduce the energy barrier, facilitating electron transfer and thereby enhancing the ML intensity. As shown in **Figure** **3**a–g, the dual‐phase heterostructure material ZnS:Mn^2+^ is incorporated into wood pulp to produce ML paper, which exhibits the yellow emission (λ = 590 nm) upon scraping. Using the UV response of the material, a dual anti‐counterfeiting label integrating static (fluorescence) and dynamic (ML) encryption capabilities is constructed. Furthermore, by combining Ho^3+^‐doped CaZnOS green ML material with ZnS:Mn^2+^, dual‐color (yellow/green) patterns are covertly printed via screen printing. These patterns do not luminesce under UV excitation but generate localized ML upon mechanical stress, significantly enhancing encryption complexity.

### 2.3 Optogenetics

**Figure 4:**
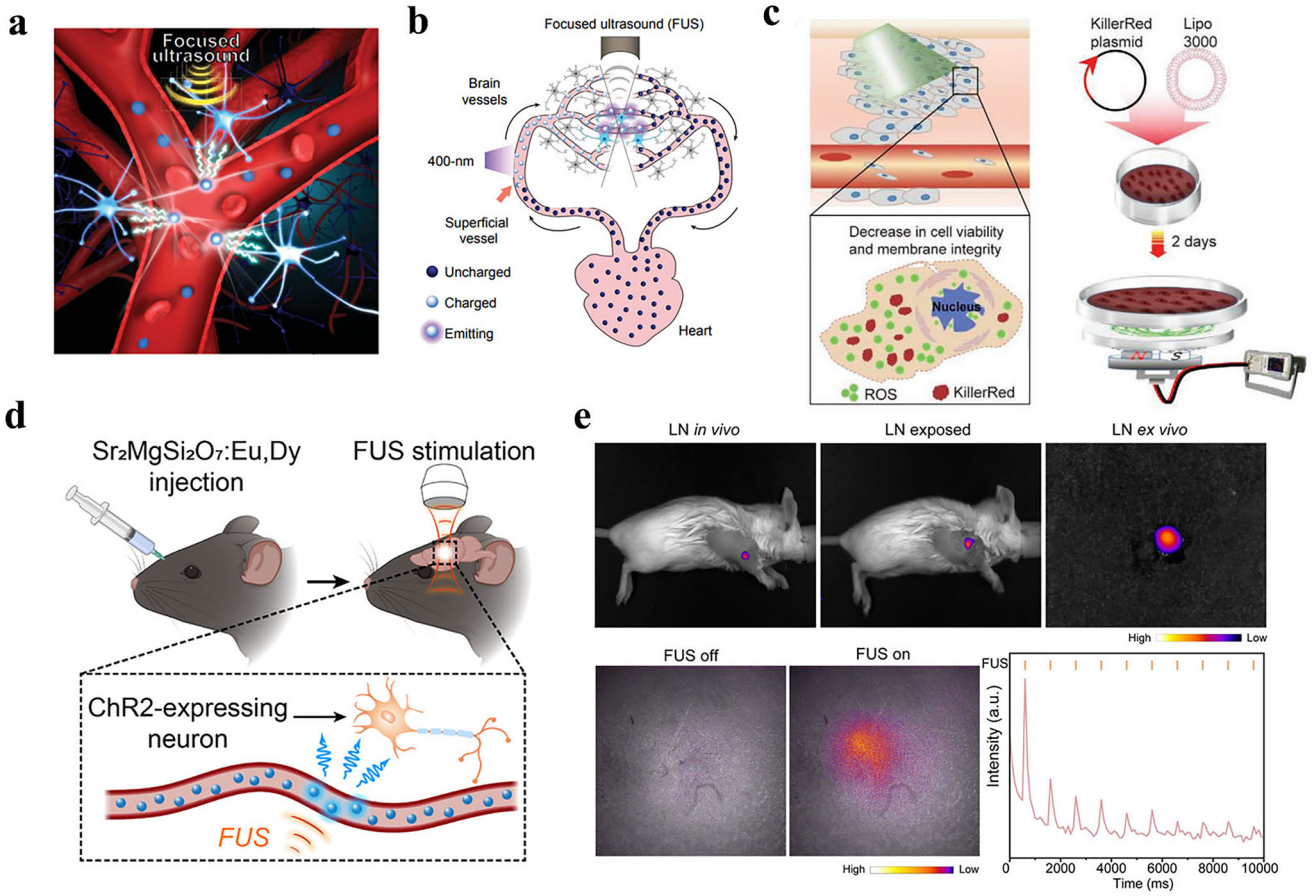
a) ZnS:Ag,Co@ZnS nanoparticles act as rechargeable light sources in blood circulation for sono‐optogenetics; b) Schematic showing blood circulation of ZnS:Ag, Co@ZnS nanoparticles, transporting the 400 nm photoexcitation energy at superficial vessels into 470 nm emission in deep‐brain regions for optogenetic stimulation, Reproduced with permission.^[^ ^37^ ^]^ Copyright 2019, National Academy of Sciences of America; c) The in vitro tumor treatment by stimulation of a non‐implantable magneto‐luminescence microdevice device, Reproduced with permission.^[^ ^60^ ^]^ Copyright 2020, Wiley‐VCH; d) Schematic showing the experimental procedure of ultrasound‐mediated optogenetic stimulation with ML colloids composed of Sr_2_MgSi_2_O_7_:Eu, Dy, Reproduced with permission.^[^ ^61^ ^]^ Copyright 2022, American Chemical Society; e) In vivo PerL imaging and FUS‐induced ML imaging in Balb/c mouse, Reproduced with permission.^[^ ^45^ ^]^ Copyright 2024, Wiley‐VCH.

Optogenetics is a technique that employs visible light to control light‐gated ion channels, enabling precise manipulation and analysis of neural circuits with neuronal subtype specificity.^[^ ^57^, ^58^, ^59^ ^]^ However, due to the limited penetration depth of visible light in biological tissues, in vivo optogenetic modulation typically requires invasive craniotomy or intracranial implantation of optical fibers. In contrast, 0D stress visualization detection technology uses ML materials to convert focused ultrasound‐induced acoustic radiation force or pressure fluctuations into variations in fluorescent signals. This enables visualization of energy distribution in the treatment area, tracking of dynamic changes in tissue stiffness, evaluation of therapeutic response, and the construction of a closed‐loop navigation system, thereby significantly enhancing the precision of diagnosis and therapy. In 2019, Hong et al. proposed a non‐invasive neuroregulation technique termed “sono‐optogenetics.”^[^ ^37^ ^]^ This approach involves intravenous injection of rechargeable ML nanoparticles (ZnS:Ag,Co@ZnS) into the mouse circulatory system. These nanoparticles can be optically “recharged” at superficial blood vessels under 400 nm light irradiation, and subsequently emit stable and sustained blue light (470 nm) when triggered by FUS (1.5 MHz), enabling precise optogenetic stimulation of neurons expressing the light‐sensitive channel protein Channelrhodopsin‐2 (ChR2) (**Figure** **4**a,b). This acousto‐optic‐based optogenetic approach enables neuronal stimulation with minimal damage to brain tissue, offering a novel strategy for non‐invasive applications of ML materials in vivo.

In 2020, Wang et al. designed two magnetically controlled ML micro‐devices (MLMDs) of different sizes by integrating CaZnOS:Tb^3+^ ML material with magnetic rods: ① A non‐implantable device (petri‐dish structure, 30–65 mm in diameter) for superficial tissue therapy, and ② an implantable device (glass capillary structure, 0.5–2 mm in diameter) for deep neural stimulation.^[^ ^60^ ^]^ Driven by an external rotating magnetic field, these micro‐devices utilize mechanical stress to induce tribo/piezoelectric potentials in the material, thereby triggering sustained green ML at 544 nm (Figure 4c). The emission intensity can be precisely modulated by varying the rotation frequency of the magnetic field (0–1600 rpm). These devices can be applied in tumor therapy and neuromodulation, respectively. For tumor treatment, the non‐implantable device is used in combination with the KillerRed protein, which exhibits an absorption spectrum ≈544 nm. Under magnetic actuation, the device induced the production of reactive oxygen species (ROS) in HeLa cells transfected with the KillerRed plasmid. In vitro experiments showed that ROS levels peaked after 15 min of stimulation, with an apoptosis rate of 49.7%, significantly higher than the 37.8% observed in the no‐light control group. In in vivo studies, intratumorally injected of KillerRed followed by treatment with the non‐implantable device resulted in a markedly smaller tumor volume (681 mm^3^) compared to the control group. Hematoxylin‐eosin (H&E) and terminal deoxynucleotidyl transferase‐mediated dUTP‐biotin nick end labeling (TUNEL) staining confirmed significant tumor cell apoptosis, while no significant change in mouse body weight is observed during treatment, demonstrating the biosafety of the device. In the field of neuromodulation, the implantable micro‐device is embedded into the motor cortex (M1) or hippocampus of mice infected with rAAV CaMKIIa‐C1V1 (E123T/T159C)‐EYFP‐WPRE‐pA (AAV‐C1V1) virus. A rotating magnetic field is applied to trigger 544 nm light emission, thereby activating the light‐sensitive channel protein C1V1. In vitro electrophysiological recordings in brain slices showed a significant increase in the frequency of spontaneous potentials in dentate gyrus neurons. During in vivo behavioral tests, free‐moving mice exhibited a 52% reduction in travel distance after M1 stimulation, with gradual behavioral recovery observed after 24 h. Immunohistochemistry analysis confirmed upregulated expression of the neuronal activation marker c‐Fos. This technology enables fully wireless operation without implanted power sources, allows precise optical control via magnetic field parameters, and minimizes invasiveness in deep tissues due to its miniaturized design (down to 500 µm). These features provide a novel toolset for photodynamic therapy and neural circuit research, particularly offering unique advantages in long‐term behavioral studies of freely moving animals. Future improvements in material efficiency and device integration may further extend its applications to cardiovascular regulation, immunomodulation, and other biomedical fields.

In 2022, Hong et al. proposed a bio‐mineral‐inspired “dissolution inhibition strategy,” mimicking the kinetic self‐protection mechanism by which biological minerals such as bones and tooth enamel resist dissolution in physiologically undersaturated environments.^[^ ^61^ ^]^ This method successfully transformed bulk ML materials synthesized via high‐temperature solid‐state reactions, including Sr_2_MgSi_2_O_7_:Eu^2+^,Dy^3+^, and ZnS:Cu,Al into stable colloidal solutions with particle diameters as small as 20 nm. This approach leverages the dissolution energy barrier resulting from high solid‐liquid interfacial tension (γ_SL_), with a critical size r* ≈10–100 nm, to achieve kinetic stabilization of nanoparticles in an undersaturated citrate solution while preserving their optical properties. As a result, the colloidal suspension emits ML intensity comparable to that of bulk materials (470–610 nm) under FUS stimulation. These ML fluids can serve as intravenous‐injectable “optical flow battery,” which are first charged via transdermal ultraviolet light (365 nm) during circulation and then triggered by tissue‐penetrating FUS to emit light at targeted deep‐tissue sites such as the brain, liver, or kidney. Furthermore, c‐fos immunohistochemical staining confirmed that FUS‐triggered ML successfully activated optogenetic effects in motor cortex neurons expressing ChR2, enabling non‐invasive neuromodulation (Figure 4d).

In 2024, Zhu et al. developed a dual‐functional Zn_2_Ga_2.96_Ge_0.75_O_8_:0.02Cr,0.02Nd@mSiO_2_ (ZGGO@MSN) nanocomposite exhibiting both NIR‐II persistent luminescence (PersL) and ML (Figure 4e).^[^ ^45^ ^]^ Using a mesoporous silica template‐confined synthesis strategy to regulate crystal size, the synthesized material achieved NIR emission at 694, 883, 1062, and 1335 nm. Coated with a dipalmitoylphosphatidylcholine (DPPC) in the immunoadjuvant R837, the composite allowed reversible disruption of the DPPC layer under FUS, enabling controlled release of R837 while simultaneously triggering NIR‐II ML from ZGGO@MSN. This established a closed‐loop regulation mechanism of “FUS triggering‐drug release‐optical feedback‐immune activation.” After subcutaneous injection, focused ultrasound responsive immunomodulator loaded nano platform (FURIN) specifically accumulated in lymph nodes, enabling non‐invasive tracking via PersL imaging. The system demonstrated excellent repeatability and biocompatibility, and could be repeatedly recharged using a 635 nm LED, supporting long‐term monitoring. This technology addresses the critical challenges of spatiotemporal precision and real‐time feedback in deep‐tissue immunomodulation, offering a precise and visual theranostic strategy for intervening in cancer metastasis and treating autoimmune diseases.

## 3 1D Stress Visualization Detection

The 1D stress visualization detection technology through dynamic monitoring of the stress distribution along a linear path, which can achieve precise detection of the mechanical behavior in a single‐axis direction. The core lies in utilizing the linear response characteristic of ML materials, and reflecting the stress gradient distribution through the variation of fluorescence intensity or wavelength along the axial direction. Currently, it is mainly achieved through two technical routes: One is to develop flexible emitting fibers, films, or optical fibers with directional stress response properties, which can generate quantifiable fluorescence signal changes when subjected to stretching/bending deformations. The other is to construct a linear sensing array, integrating discrete ML units at specific intervals on a flexible substrate. With the development of micro‐nano manufacturing technology, 1D stress visualization detection is evolving toward higher spatial resolution and faster response speed, providing innovative technical means for engineering safety assessment and biomechanical research.

### 3.1 Electronic Signatures

**Figure 5:**
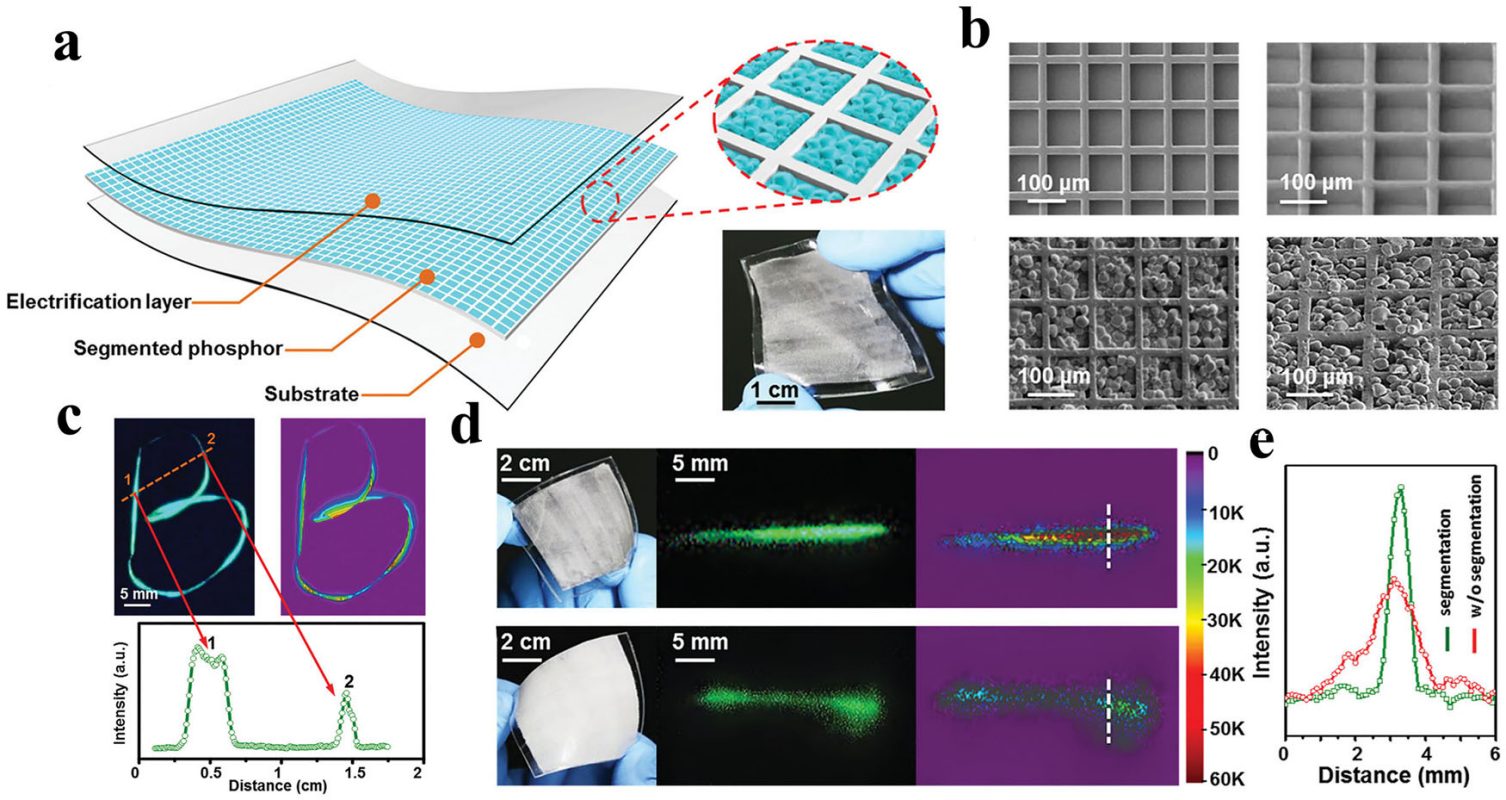
a) Schematic diagram of the composite material with the segmentation structure in the luminescent layer, inset: magnified view of the segmentation structure; b) Photograph of a twisted composite material; c) Superimposed image showing the complete trajectory of a letter “B,” and ML intensity distribution along the dashed line; d) the trajectory corresponding mapping of the ML intensity; e) ML intensity distribution along the dashed lines, Reproduced with permission.^[^ ^47^ ^]^ Copyright 2016, Wiley‐VCH.

1D stress visualization detection enables dynamic monitoring of stress distribution along a linear path, allowing precise capture and real‐time visualization of dynamic mechanical characteristics during the writing process, thereby effectively preventing potential forgery. In 2016, Zhu et al. designed a multilayer flexible composite for electronic signature applications based on the triboelectrification‐induced electroluminescence (TIEL) mechanism, where gentle mechanical interactions are directly converted into optical signals. The device architecture featured a 100 µm polyimide substrate, a luminescent layer of ZnS:Cu particles (≈20 µm) embedded in PMMA, and a 250–50 µm thick FEP copolymer layer serving as the triboelectric component.^[^ ^47^ ^]^ As shown in **Figure** **5**a,b, to enhance the spatial resolution of motion tracking, a square microcavity array with a depth of 45 µm, side length of 100 µm, and interval of 20 µm is etched onto a silicon wafer using photolithography. The microcavities are filled with graphite‐doped PDMS to form isolation walls. After peeling off, a flexible microcavity template is obtained. A mixture of ZnS:Cu/PMMA ML material is then filled into the microcavities and cured at 130 °C to form independent light‐emitting units. The light blocking property of the graphite‐PDMS walls effectively prevents optical crosstalk between adjacent units. When a heterogeneous material slides against the triboelectric layer, the rapid accumulation and redistribution of interfacial triboelectric charges instantaneously excite electroluminescence from the underlying phosphors. This composite exhibits an ultralow threshold stress of 10 kPa and a responsivity of 0.03 kPa^−1^, 750 times higher than conventional triboluminescence (TL), while maintaining stable emission without requiring a vacuum environment. As shown in Figure 5b,c, the grid‐structured design of the light‐emitting layer effectively suppresses optical crosstalk, enabling motion trajectory visualization with high spatial resolution (1 mm). Integrated with an image acquisition system and software processing, the system successfully achieves real‐time handwritten trajectory recording (e.g., the letter “B”), intensity distribution mapping, and large‐area luminescence modulation. It accurately captures dynamic parameters such as sliding speed and contact stress, offering an innovative solution for security monitoring, anti‐counterfeiting in electronic signatures, and human‐machine interfaces. Furthermore, the high stability of the TIEL, less than 5% intensity degradation after 20 000 cycles, and its bending tolerance (less than 5% fluctuation at a curvature of 100 m^−1^) underscore its potential for flexible wearable devices and curved surface sensing applications.

### 3.2 Transparent Glass‐Optical Fiber Stress Sensing

**Figure 6:**
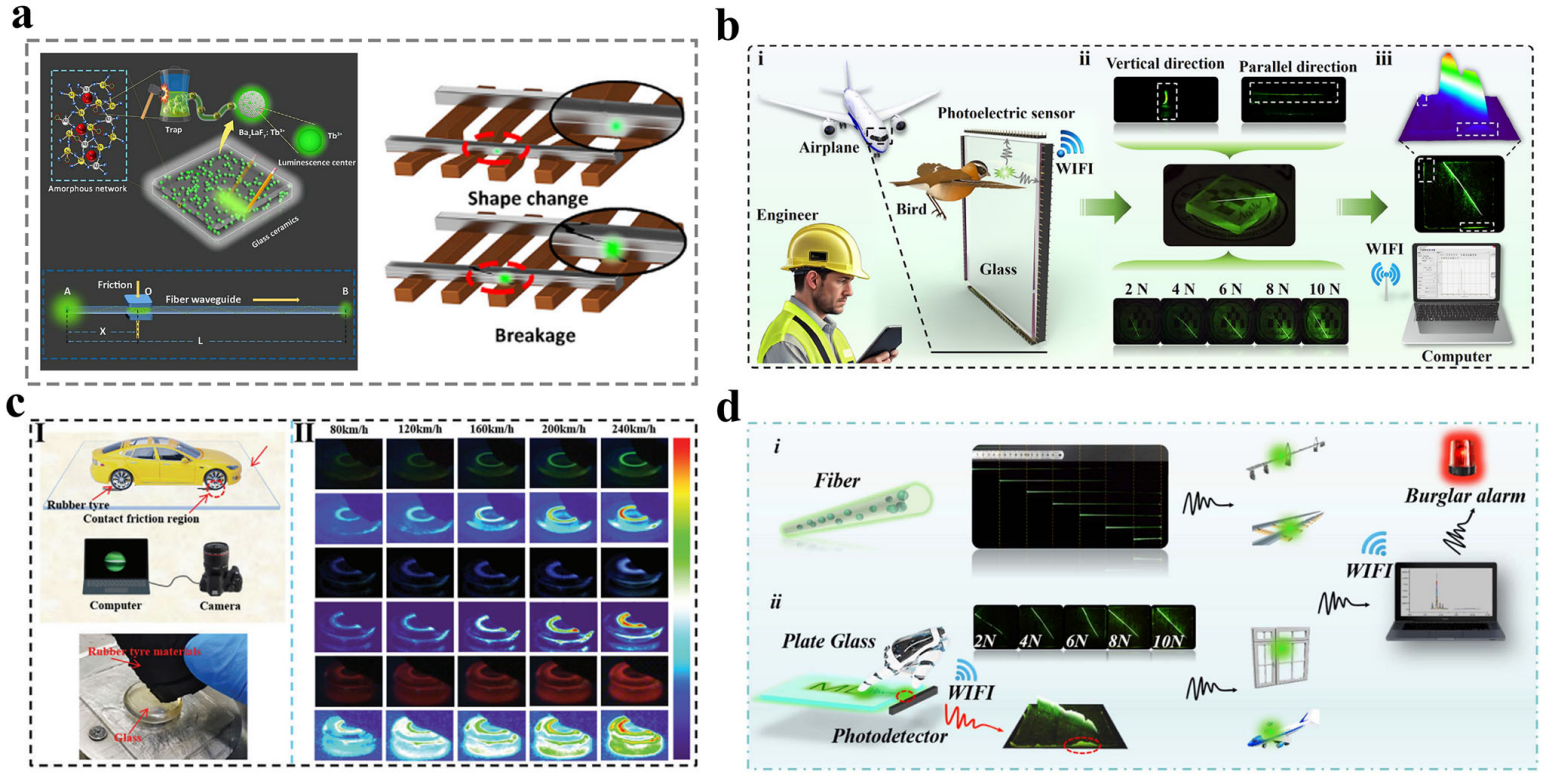
a) Schematic of the as‐developed stress sensor system, Reproduced with permission.^[^ ^46^ ^]^ Copyright 2022, Cell Press; b) Schematic diagram of application to stress safety monitoring of aviation aircraft, Reproduced with permission,^[^ ^62^ ^]^ Copyright 2023, Wiley‐VCH; c) Diagram of potential application of CaF_2_:Tb^3+^ GCs, Reproduced with permission.^[^ ^63^ ^]^ Copyright 2023, Royal Society of Chemistry.

The transparent glass‐fiber optic sensing system leverages the high transparency of glass and it waveguiding properties. When external stress is applied to the glass surface and transmitted along a 1D path into the optical fiber, it induces changes in the light transmission characteristics within the fiber. These optical responses are synchronously recorded and directly mapped into high spatial resolution stress intensity and location information, which is displayed in real time as a continuous curve (light intensity vs distance) along a 1D axis. This visualization capability allows researchers to directly “see” stress concentration points, stress gradients, and propagation paths without the need for complex inversion, providing an intuitive and dynamic mechanical monitoring tool for structural health diagnosis. In 2022, Xu et al. employed a controlled crystallization process to uniformly precipitate Ba_2_LaF_7_:Tb^3+^ nanocrystals with an average size of ≈18.2 nm within a glass matrix.^[^ ^46^ ^]^ By using a femtosecond laser as an excitation light source to fulfill the constructed traps, they endowed the material with excellent ML properties. A self‐driven optical fiber sensor for remote stress detection has been subsequently developed utilizing the material. Under mechanical stimulation, carriers released from the traps recombine at the Tb^3+^ emission center, triggering bright green ML peaking at 545 nm. The emission intensity exhibits a linear response to applied force (R^2^ >0.99) and maintains a high signal‐to‐noise ratio even under daylight conditions. The high visible light transmittance (>80%) and waveguiding properties of the glass‐ceramic enable efficient remote transmission of ML signals through the optical fiber. As shown in **Figure** **6**a, a dynamic stress trajectory imaging test is conducted using a 2 m long optical fiber. By analyzing the ratio of light intensities at both ends, the system achieved precise localization of both the position and magnitude of stress with an error of less than 5%. Compared to conventional electronic sensors, this stress‐sensing device requires no external power supply or complex demodulation systems, and offers advantages such as immunity to electromagnetic interference and robustness in harsh environments. Furthermore, the strategy has been successfully extended to other fluoride glass systems such as NaYF_4_ and LaF_3_, demonstrating the general applicability of glass‐ceramic materials in stress sensing.

In 2023, Liu et al. successfully prepared TbBO_3_ glass‐ceramics via a two‐step melt‐quenching method.^[^ ^62^ ^]^ By leveraging the high structural stability of silicon–oxygen (Si–O) and boron–oxygen (B–O) tetrahedra within the glass matrix, along with femtosecond laser‐induced ultra‐deep traps (517 K), they achieved stress‐responsive properties with strong environmental resistance and have the PersL performance. Femtosecond laser irradiation generated a non‐bridging oxygen vacancy center in the glass, which act as an efficient charge carrier traps. Under mechanical stress, trapped carriers are released and migrate to the Tb^3+^ emission center, resulting in intense ML peaking at 546 nm, corresponding to the ^5^D_4_→^7^F_5_ transition of Tb^3+^. The ML intensity exhibits a linear response to applied pressure (R^2^ >0.99) and remains clearly visible even under daylight conditions. The material retains over 70% of its initial ML intensity after 21 days of storage and demonstrates excellent environmental stability, with no significant luminescence degradation after immersion in air, water, or alcohol for up to 10 h. Thanks to its large Stokes shift (268 nm) and negligible self‐absorption, the ML signal can be remotely transmitted via optical waveguide effects. When combined with a linear attenuation model, this enables precise spatial localization of stress points. Based on this, the research team developed an aircraft windshield stress safety monitoring system. As illustrated in Figure 6b, when a bird or foreign object impacts the windshield, the resulting ML signal is transmitted through the glass to edge‐mounted photoelectric sensors. The impact location is then wirelessly communicated in real time, providing a visualized monitoring solution for flight safety.

As shown in Figure 6c, Zhao et al. reported a multimodal ML system based on CaF_2_:Tb^3+^glass‐ceramics.^[^ ^63^ ^]^ By incorporating oxygen vacancy defects as deep traps (1.044 eV) within the rigid transparent glass‐ceramic matrix, and utilizing friction‐induced thermal excitation to release trapped carriers, green ML emission centered at 546 nm is achieved. The ML intensity exhibits a linear response to applied stress (R^2^ = 0.99). Moreover, the material retains over 70% of its initial emission intensity after 21 days of storage, demonstrating exceptional stability. In flexible applications, the CaF_2_:Tb^3+^ glass‐ceramic (GCs) powder is composited with PDMS to achieve self‐recovering ML under various mechanical stimuli such as stretching and folding. This is realized through inorganic–organic interfacial friction‐induced electric potential, which drives electron bombardment to directly excite Tb^3+^ emission centers. Furthermore, by doping with ions such as Eu^3+^ and Eu^2+^, the red, blue, and green ML emission are achieved, extending the full‐spectrum stress visualization capability of the glass‐ceramic system. In rigid media, the ML signal can be remotely transmitted via the optical waveguide effect. By analyzing the optical intensity ratio from both ends, the stress location can be accurately determined, enabling the design of systems such as aircraft windshield impact monitoring. Meanwhile, flexible composite materials show promise for developing an “optical skin” that monitors human joint motion in real time, with wirelessly transmitted feedback on biomechanical data.

### 3.3 Clinical Medical Monitoring

**Figure 7:**
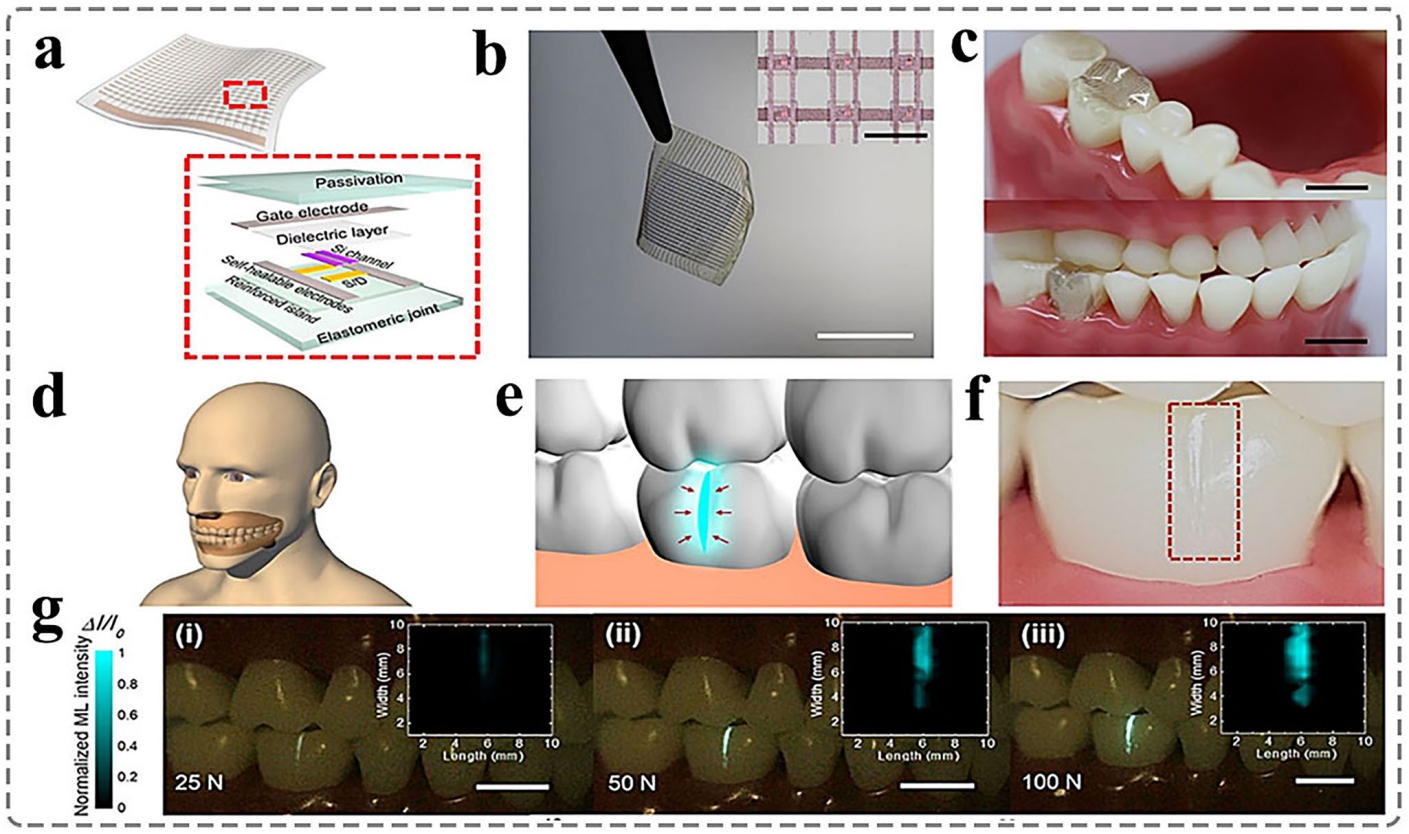
a) Schematic illustration and b) Photographs of a 20×20 array of FETs with stretchable and self‐healable interconnections fabricated on the rigid‐soft hybrid substrate. The scale bar is 1 cm. Inset shows an optical micrograph image of this PD array; The Black scale bar is 500 µm; c) Photographs of the stretchable PD array laminated on the top surface (occlusal) and facial surface (buccal) of the molar teeth. Scale bars are 1 cm; d) Schematic illustrations of the mechanism for light emission of ZRT phosphor particles in cracked areas. e) Photographs of denture including tiny crack filled with ZRT phosphor particles; f) Red arrows indicate the concentration of the force; g) Photographs showing the occurrence of ML from the crack of the molar tooth by the compressive force of (i) 25, (ii) 50, and (iii) 100 N. The corresponding inset images show 2D mapping images of ML emission using a PD array. Scale bar is 1 cm in all the cases, Reproduced with permission.^[^ ^66^ ^]^ Copyright 2022, Springer Nature.

1D stress visualization technology holds potential for clinical medical monitoring, such as real‐time detection of stress distribution along the tooth surface or a predefined internal path via miniature mechanical sensors. This system translates the mechanical behavior of crack propagation into intuitive visual data.^[^ ^64^, ^65^ ^]^ When external force (e.g., occlusal load) is applied to the tooth, a characteristic stress concentration zone forms around the crack tip. A sensor network deployed along a 1D detection axis (aligned with the potential direction of crack propagation) measures continuous spatial variations in strain/stress, generating high‐resolution 1D stress distribution profiles. The acquired data are then visualized through pseudo‐color coding or dynamic waveform graphs overlaid onto a 1D axial coordinate model of the tooth. This enables clear identification of crack location, depth, and activity, providing non‐destructive stress imaging support for clinical diagnosis. In 2022, Kim et al. reported an innovative approach for detecting tooth cracks based on the synergistic use of the ML material ZrO_2_:Ti^4+^ (ZRT) and a stretchable photodetector (PD) array.^[^ ^66^ ^]^ This technology aims to overcome limitations of conventional methods, such as transillumination and X‐ray imaging in diagnosing cracked tooth syndrome (CTS), including low resolution, radiation risks, and the inability to distinguish micron‐scale cracks from superficial scratches. By synthesizing a biocompatible ML material, ZRT, the system leverages its ability to release stored carriers through a trap under external forces (e.g., chewing pressure), and then the carriers transfer to the emission center and emit cyan blue light peaking at 477 nm. When integrated with a flexible and self‐healing PD array, this mechanism enables real‐time, non‐invasive, and high‐resolution imaging of microcracks. As illustrated in **Figure** **7**, ZRT particles are applied into tooth cracks. Under chewing force, they exhibit a localized stress amplification effect, which can activate the material's ML emission. The system utilizes ZRT biocompatibility and high mechanical strength to achieve stable emission in the oral environment. The developed stretchable and self‐healing PD array combines high photosensitivity (response time: 10 ms, recovery time: 15 ms), mechanical durability, and resistance to oral environmental interference, overcoming the limitations of conventional rigid devices in conforming to curved tooth surfaces. A dynamic stress‐responsive ML signal acquisition mechanism has been introduced, where luminescence is triggered only under external force, allowing effective discrimination between functional cracks and non‐pathological scratches. Experimental results demonstrate that this method provides clear imaging of microcracks under chewing forces ranging from 25 to 100 N, without the need for complex equipment or harmful radiation. It offers a highly sensitive, low‐cost, and non‐invasive diagnostic solution for early‐stage CTS, marking a groundbreaking application of ML technology in biomedical sensing.

### 3.4 Dual‐Mode Sensing Based on Temperature and Stress

**Figure 8:**
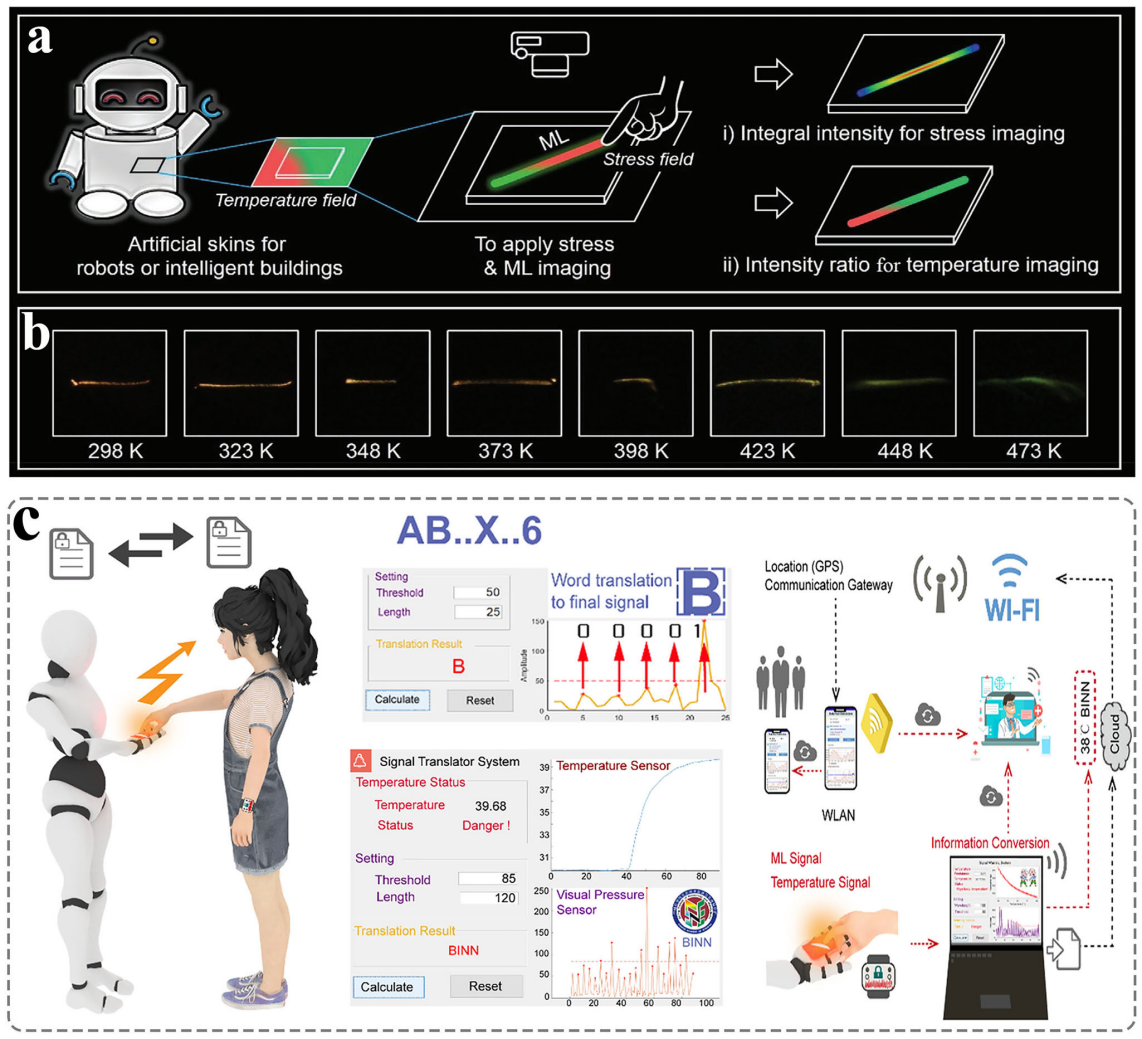
a) Application of the stress‐temperature dual‐modal imaging based on the double‐lanthanide‐activated ML materials; b) Photographs of the ML film being scraped at different temperatures, Reproduced with permission.^[^ ^70^ ^]^ Copyright 2021, Wiley‐VCH; c) Bimodal sensor for user‐interactive applications, Reproduced with permission.^[^ ^71^ ^]^ Copyright 2022, American Chemical Society.

The 1D stress visualization detection technology enables dual‐mode sensing of both temperature and stress by using the distinct response characteristics of ML materials to thermal and mechanical stimuli.^[^ ^67^, ^68^, ^69^ ^]^ In 2021, Xie et al. developed a ML material, SrZnOS:0.02 Tb,0.01Eu, co‐doped with dual rare earth ions. By precisely controlling the doping ratio of the rare earth ions, they successfully achieved a material exhibiting high sensitivity to both stress and temperature.^[^ ^70^ ^]^ As shown in **Figure** **8**a, a ML film is fabricated by integrating SrZnOS:0.02 Tb,0.01Eu ML particles into an organic polymer matrix. This film serves as an intelligent e‐skin, conformally attached to robotic surfaces to detect external mechanical stress or temperature variations. When subjected to mechanical stimuli, the resulting ML is captured as an intensity distribution. The absolute ML intensity can be used to quantify stress magnitude, while the ratio of emission intensity at different wavelengths enables temperature sensing. Figure 8b illustrates the variation in ML color of the film as the temperature increases gradually from 298 to 473 K, showing a clear transition from orange to green emission. This result demonstrates the feasibility of using dual rare‐earth‐activated ML materials for dual‐mode sensing of both stress and temperature. Furthermore, tactile sensors with multi‐modal sensing capabilities form the foundation for the application of biomimetic skin in humanoid robots and intelligent prosthetics.

Pan et al. utilized the stress‐to‐light conversion properties of ML materials to develop a temperature and pressure‐sensitive tactile sensor that does not require signal fusion.^[^ ^71^ ^]^ The sensor integrates photonic and electronic sensing mechanisms, enabling independent detection of both temperature and pressure, while eliminating the need for signal separation algorithms and computational processing. As shown in Figure 8c, the dual‐mode sensor consists of ML powder (ZnS‐CaZnOS) and thermally resistant organic polymers, which convert pressure and temperature into optical and electrical signals, respectively. It demonstrates a temperature sensitivity of −0.6% °C^−1^ within the range of 21–60 °C and is capable of stress sensing under pressures as low as 2 N. Owing to its non‐interfering optical properties, this sensor enables encrypted communication, temperature and pressure monitoring, and wireless signal transmission in robotic applications. This work offers a novel strategy for mitigating signal interference in multimodal tactile sensing systems.

## 4 2D Stress Visualization Detection

**Figure 9:**
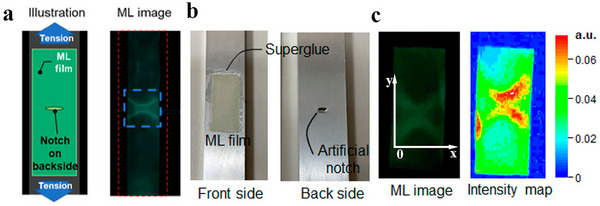
a) Changes in the intensity distribution of ML paint on the cracked composite specimen over time during tensile tests; b) an aluminium alloy specimen; c) ML intensity test with an aluminium alloy specimen, Reproduced with permission.^[^ ^72^ ^]^ Copyright 2022, American Chemical Society.

The 2D stress visualization detection technology by presenting the stress distribution within the plane, which can achieve a comprehensive characterization of complex stress fields. This detection technology is mainly based on the biaxial stress response characteristics of ML materials. By capturing the ML intensity of the material at different positions within a plane, a 2D stress distribution map is constructed (**Figure** **9**).^[^ ^72^ ^]^ Currently, two main implementation paths are adopted: One is to prepare flexible ML film materials, and then reflect the direction and magnitude of the principal stress through the stress‐induced anisotropic ML changes. Second, a high‐density sensing array is constructed. Using micro‐nano processing technology, ML units are integrated on the flexible substrate to achieve a discrete measurement of the in‐plane stress distribution. This stress visualization detection technology can be applied in fields such as damage assessment of composite materials, reliability testing of flexible electronic devices, and visualization analysis of forces during motion. With the ongoing advancement of ML materials, 2D stress visualization technology is progressing toward greater intelligence and higher precision. It is expected to achieve submicron spatial resolution and millisecond‐level dynamic response in the future, thereby delivering more powerful stress analysis tools for applications such as advanced manufacturing and tactile sensing.

### 4.1 Damage Detection

#### 4.1.1 Visualization Detection of Microcracks in Bridges

**Figure 10:**
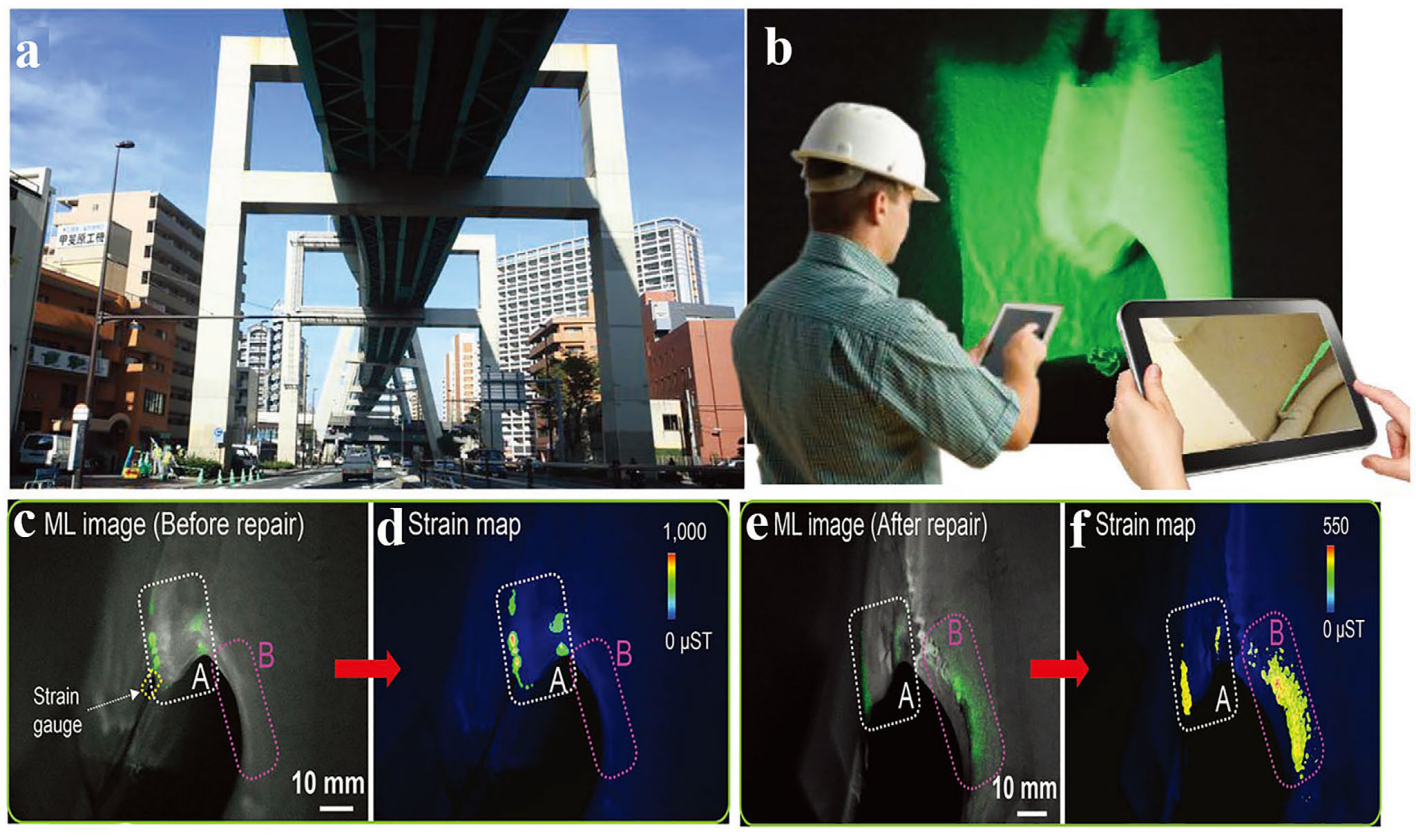
a) Outside view of the inspected infrastructure (urban expressway); b) Future image of onsite inspection on infrastructure; Images of ML c) and strain d) show a high stress concentration with a maximum strain of ≈1000 µST before repair; Images of ML e) and strain f) show the above concentration with a maximum strain down to ≈500 µST after repair (grinding the welding section using a grinder), Reproduced with permission.^[^ ^73^ ^]^ Copyright 2018, Wiley‐VCH.

2D in‐plane stress visualization technology is based on the visualization of stress distribution through ML materials, enables real‐time monitoring of stress concentration and damage evolution in composite laminates for engineering applications. In 2018, Xu et al. proposed a stretchable strain sensor based on the mechanism of elastic ML. Utilizing the repeatable ML of SrAl_2_O_4_:Eu (SAOE) material under elastic deformation, the sensor enables dynamic stress/strain imaging across multiple scales from micrometers to meters.^[^ ^73^ ^]^ The sensor takes the form of a smart coating, composed of SAOE microparticles (average particle size ≈1 µm) dispersed in a transparent epoxy matrix. It can be applied to flat or complex curved surfaces via screen printing or spray coating. Under tensile loading, the resulting ML is captured by a CCD camera. The accuracy of strain distribution is quantitatively validated through simultaneous strain gauge measurements and finite element simulations. As illustrated in **Figure** **10**, the sensor is applied via on‐site spraying at the U‐rib connections of a steel box girder in an urban viaduct. Dynamic loads from passing vehicles excite ML, enabling a quantitative assessment of stress concentration. Post‐repair measurements confirmed a 40% reduction in stress concentration, demonstrating the viability of this technology for real‐time hazard classification and maintenance evaluation across large‐scale infrastructures. Furthermore, the system is used to achieve dynamic optical imaging on a simulated femur under biomechanical loading, and emission patterns generated by finger pressure on a sensor‐coated spherical surface highlighted its potential in biomechanical and curved‐surface monitoring. All applications rely on the sensor's ability to emit light under elastic deformation. Using optical imaging systems and quantitative calibration methods, the optical signals are converted into precise strain distribution maps, enabling dynamic, full‐field, and quantitative stress visualization for monitoring complex structures and practical engineering scenarios.

#### 4.1.2 Visualization Detection Based on Absolute ML Intensity

**Figure 11:**
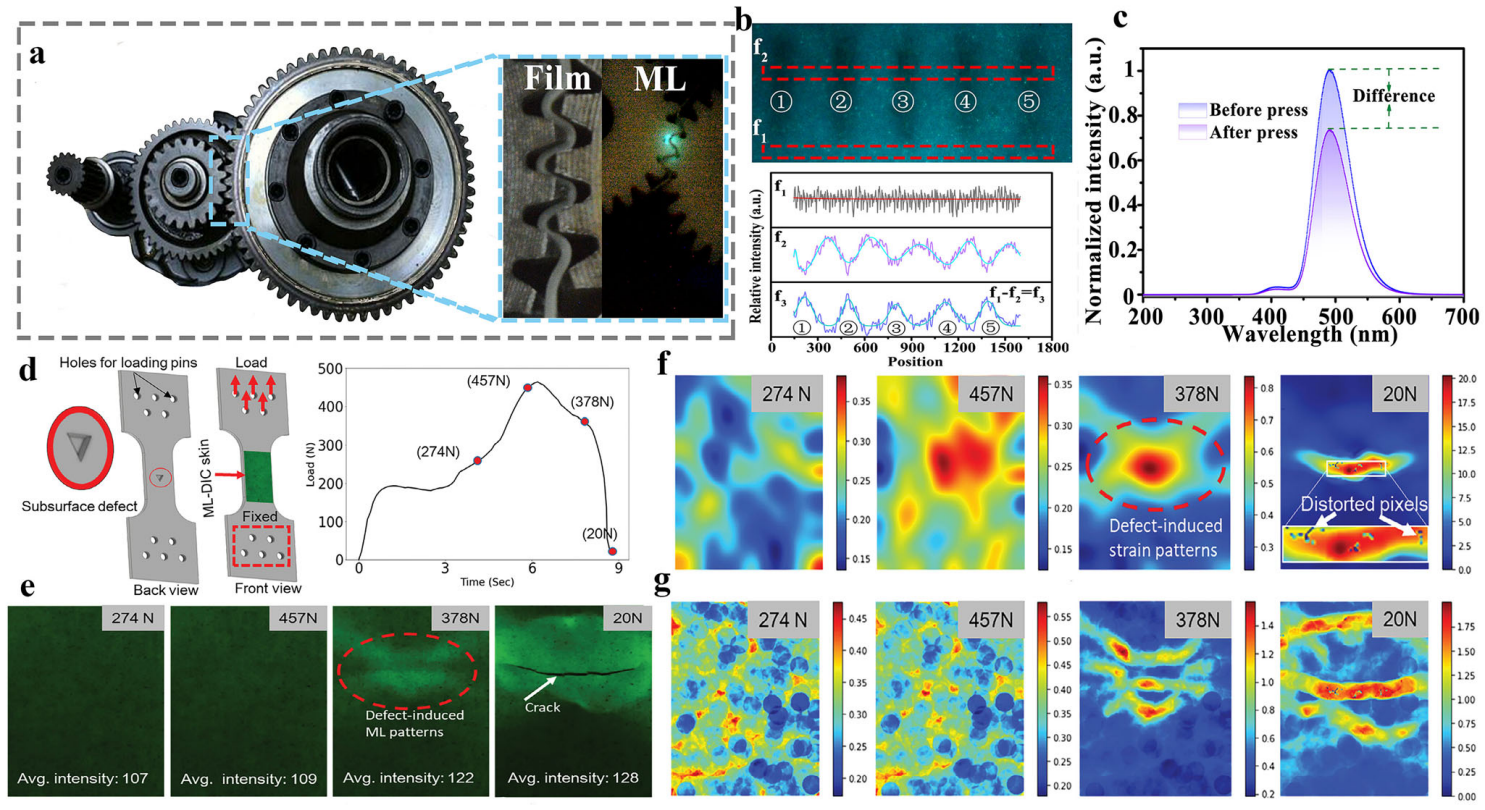
a) Motorcycle internal gear component illustration and ML originating from the gear occlusion; b) Digital picture of the SAOET/PDMS elastomer with 980 nm laser diode irradiation after mechanical stimulation and the corresponding integrated intensity profile derived from f_1_ and f_2_, showing the stress memory intensities corresponding to A, B, C, D and E by f_1_‐f_2_; c) PSL spectra of the SAOET/PDMS film before and after the gear occlusion, Reproduced with permission.^[^ ^74^ ^]^ Copyright 2022, Royal Society of Chemistry; d) Tension specimen with a subsurface defect of triangular geometry alongside the load curve; e) Photographs corresponding to the load levels; f,g) The normalized least‐squares criterion (NLSC) mappings under different load, Reproduced with permission.^[^ ^33^ ^]^ Copyright 2024, Wiley‐VCH.

Owing to mechanical wear during operation, complex structural components generally exhibit a limited service life. Monitoring the wear condition and operational status of these components is essential to ensure the safety of machinery. 2D in‐plane stress visualization technology can provide an intuitive display of stress distribution in the working regions of such parts. In 2022, Xu et al. developed a flexible ML film with stress memory functionality based on the trap‐controlled ML material Sr_4_Al_14_O_25_:Eu^2+^,Tm^3+^.^[^ ^74^ ^]^ During the operation of mechanical components, the trapped carriers are released in regions under contact stress, while those in unstressed areas remain confined within the traps. By analysing the distribution of trapped carrier concentration, this method enables monitoring of mechanical behavior in complex components, such as the stress distribution during gear engagement. As shown in **Figure** **11**a–c, the ML film is fabricated by incorporating the trap‐controlled material Sr_4_Al_14_O_25_:Eu^2+^,Tm^3+^ into a PDMS polymer. This ML film is attached to the engagement area of two meshing gears for stress testing. When the gears engage during operation, mechanical stress compresses the ML film. The compressed regions emit visible ML due to the release of trapped carriers from traps under stress. In contrast, carriers in unstressed regions remain stored in deeper traps, enabling long‐term stability. The stress magnitude applied to the gears can be quantified based on the energy difference between regions with released and unreleased carriers. Although no visible change is observed in the film after compression, the distribution of trapped carriers is altered. A comparison of photo‐stimulated luminescence spectra before and after stress exposure reveals clear differences in emission intensity, demonstrating a linear stress‐memory effect within the ML film corresponding to the applied pressure.

In 2024, Kim et al. developed a dual‐modal ML digital image correlation (DIC) smart sensing skin, composed of SrAl_2_O_4_:Eu,Dy (SAO) ML particles, acrylic resin, and carbon nanotubes (CNTs).^[^ ^33^ ^]^ This composite system enables synchronous full‐field deformation measurement via DIC and ML response monitoring under various illumination conditions, including white LED light (WLED), darkness (UV‐OFF), and ultraviolet light (UV‐ON). As shown in Figure 11d–g, the ML‐DIC skin is bonded to the surfaces of an epoxy resin tensile specimen, a compact tensile shear (CTS) crack specimen, and an aluminum alloy specimen with a subsurface triangular defect. Cyclic loading is applied using a universal testing machine, while a CCD camera recorded the deformation process. In dark environments, DIC imaging is performed using the PersL of SAO. Under UV illumination, photoluminescence (PL) is utilized. While under white light, imaging relied on reflected light and CNT speckle patterns. The DIC software computed displacement and strain fields in real time, and the stress intensity factor (SIF) is further determined through inversion of the crack‐tip displacement field with an accuracy of 0.02–0.04 MPa√m. Meanwhile, the ML emission distribution showed significant agreement with the effective strain field, particularly revealing clear strain concentration patterns in crack propagation zones and near subsurface defects. This integration enables simultaneous defect visualization and quantitative strain field measurement. The system achieves high‐precision, full‐field, and real‐time strain monitoring and defect diagnosis under various lighting conditions using only a single camera, without the need for complex optical setups. This provides a highly adaptable solution for structural health monitoring in engineering applications and the study of mechanical behavior in materials.

#### 4.1.3 Visualization Detection Based on the Ratio of ML Intensity

**Figure 12:**
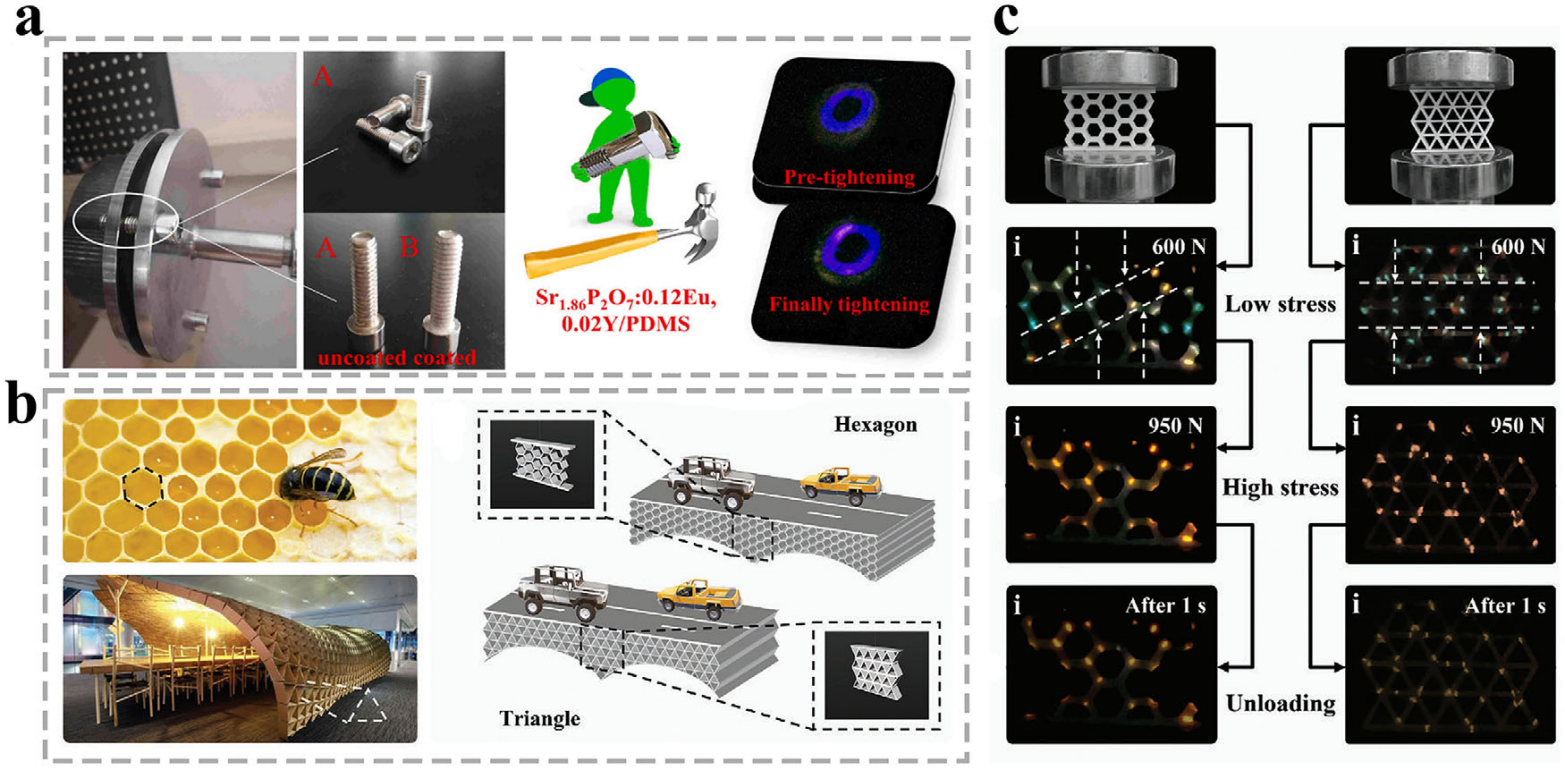
a) Application demonstration for the visualized semi‐quantitative sensing of contact mechanics, Reproduced with permission.^[^ ^78^ ^]^ Copyright 2014, Elsevier; b) Polygonal structures in honeycomb and artificial architecture and hexagon and triangular bridge structures; c) ML characteristics of the printed hexagonal structure and triangle structure under different loads, Reproduced with permission.^[^ ^79^ ^]^ Copyright 2023, Wiley‐VCH.

With the continued advancement of ML materials, researchers have incorporated a dual emission center into the material design, enabling self‐calibrating stress measurement based on changes in emission color.^[^ ^75^, ^76^, ^77^ ^]^ This approach significantly enhances environmental robustness against interference. Furthermore, 2D stress visualization techniques allow detailed analysis of the in‐plane stress distribution under varying mechanical conditions. In 2021, Wang et al. reported that Sr_2_P_2_O_7_:Eu,Y exhibits stress‐dependent ML color variation.^[^ ^78^ ^]^ In this material, both Eu^2+^ (blue emission) and Eu^3+^ (red emission) ions coexist. By co‐doping with Y^3+^ ions, the ratio between Eu^2+^ and Eu^3+^ can be effectively modulated. Owing to their distinct stress response sensitivities, the material demonstrates noticeable luminescence color changes under mechanical stimuli. As shown in **Figure** **12**a, they prepared a flexible composite film by integrating Sr_2_P_2_O_7_:Eu,Y ML particles into PDMS. Under tensile strains ranging from 20% to 60%, the film exhibits blue ML. As the strain increases to 60–100%, the emission shifts to purple. Using this stress‐dependent color variation, they proposed a semi‐quantitative visual stress‐sensing technology based on the developed ML film. By recording the ML color distribution across the film under mechanical load, the local stress or strain magnitude can be visually evaluated. For instance, when applied to screw fastening points or mechanical joints, the color shift of the ML film during the tightening process can distinguish between pre‐tightening and full‐tightening stages. This capability helps prevent over‐tightening‐induced stress accumulation, thereby mitigating premature mechanical failure and enhancing equipment protection.

Based on the aforementioned stress‐dependent color variation phenomenon, Yu et al. developed the dual‐activator co‐doping strategy by incorporating Yb^2+^ and Mn^2+^ ions into an MgF_2_ matrix.^[^ ^79^ ^]^ Using the distinct response characteristics of the two ions to crystal field changes, the material achieves controllable changes in emission color under varying stress conditions. Under low stress conditions, the cyan emission (480 nm) dominated by Yb^2+^ is more pronounced. As stress increases, the orange emission (585 nm) from Mn^2+^ gradually intensifies, resulting in a continuous and tunable shift in ML color from cyan to orange. Using this stress‐dependent optical response, the material is integrated with UV‐curable 3D printing technology. By precisely controlling printing parameters and material composition, architectural structural models such as hexagons and triangles are successfully fabricated. As shown in Figure 12b,c, applying pressure to these 3D‐printed ML structures allows intuitive visualization of stress distribution. In the hexagonal structure, diagonally oriented stress concentration zones first exhibit orange emission, consistent with finite element simulation results. The triangular structure, in contrast, demonstrates more uniform stress distribution. This technological breakthrough enables the adaptation of such materials to structural components of arbitrary shapes and sizes, offering a novel solution for health monitoring in engineering structures.

### 4.2 Flexible Wearables

**Figure 13:**
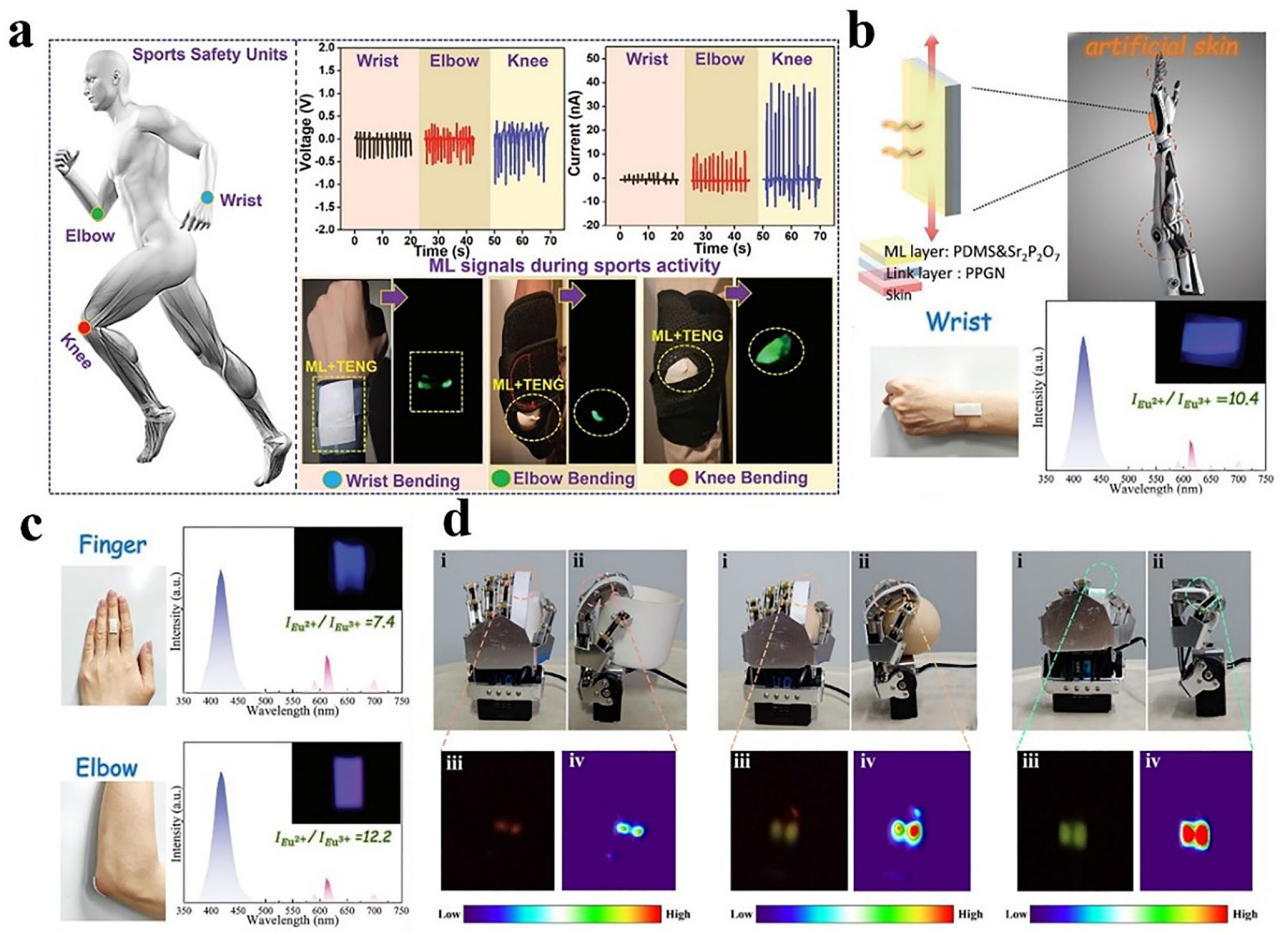
a) Illustration of installing the integrated ML and TENG device for sports monitoring and safety, the corresponding ML photos and signals generated during wrist bending, elbow bending, and knee bending, Reproduced with permission.^[^ ^84^ ^]^ Copyright 2024, Wiley‐VCH; Schematic illustration of prepared ML‐PPNG‐based artificial skin b), and the prepared artificial skin can be used to detect motion of different part human body: finger, elbow, and wrist c), the corresponding ML spectra are demonstrated and insets exhibit the ML photographs, Reproduced with permission.^[^ ^85^ ^]^ Copyright 2022, Wiley‐VCH; d) The photograph of a mechanical hand grabs the crucible, egg, and centrifuge tube, and the corresponding optical photograph vs stress distribution image, Reproduced with permission.^[^ ^86^ ^]^ Copyright 2024, Wiley‐VCH.

Flexible wearable devices can monitor human motion through optical signals generated by functional materials. These systems hold broad application potential in motion recognition, smart textiles, and monitoring physiological signals.^[^ ^80^, ^81^, ^82^, ^83^ ^]^ Depending on the target physical stimuli, such as force, temperature, humidity, light, magnetic fields, or gas, wearable smart devices can convert physical information into real‐time, visualized optical signals, enabling 2D in‐plane stress visualization and detection. In 2024, Kim et al. developed an integrated sensing system combining a single‐electrode mode triboelectric nanogenerator (TENG) and ML based on a PDMS‐ZnS:Cu composite.^[^ ^84^ ^]^ The system simultaneously generates electrical energy and visible light signals under mechanical stimulation, which can be successfully applied in various self‐powered safety monitoring scenarios. The composite is prepared by mixing ZnS:Cu particles with PDMS at a weight ratio of 7:3. Silver nanowires (Ag NWs) are embedded into the surface via spin‐coating to serve as electrodes. Under a 5 N compressive force applied by a linear actuator, the device exhibited an output of 60 V in voltage, 395 nA in current, and 15 nC in charge. As shown in **Figure** **13**a, the device is attached to human joints such as the elbow, wrist, and knee, where it generates both TENG electrical signals and ML optical signals in real time through bending motions for sports safety monitoring. In underwater environments, the encapsulated device remains functional, effectively emitting ML and producing electrical signals when bent, enabling underwater SOS distress signaling. In an application for deep‐well mining helmets, the device can be mounted on the helmet surface. When objects of different weights are dropped from a height of 30 cm, the TENG signal intensity distinguished impact energy levels (higher voltage output under heavier impacts). An artificial neural network is employed to classify the signals with an accuracy of 95.2%, while the ML provided simultaneous visual warning. Additionally, the system can generate single and double pulse sequences in the TENG output corresponding to the “dot” and “dash” signals in Morse code, enabling the encoding and transmission of messages such as “SOS.” This integrated TENG‐ML design operates without an external power supply, simultaneously providing both electrical and optical signals through mechanical energy conversion, thereby offering a real‐time, self‐powered dual‐mode sensing solution for applications such as motion monitoring, underwater operations, and mining safety.

In 2022, Wang et al. realized ratio‐based ML using a dual‐activator co‐doping strategy involving rare‐earth ions exhibiting both f‐f and f‐d transitions Figure 13b,c.^[^ ^85^ ^]^ The difference in excited‐state electronic configurations induced by various activators (e.g., the 5d‐4f transition of Eu^2+^ is sensitive to local crystal field perturbations, while the f‐f transitions of Dy^3+^/Tb^3+^ are less affected by shielding effects) leads to a linear dependence of ML intensity ratio (MLIR) and applied stress. This enables stress visualization through changes in emission color (e.g., blue‐white, green‐cyan, pink‐purple). The ratio‐based ML material quantitative evaluation system is established, defining absolute sensitivity (S_a_═∂(MLIR)/∂F, reaching up to 7.60 N^−1^), relative sensitivity (S_r_═(1/MLIR) × ∂(MLIR)/∂F × 100%, reaching up to 1.550% N^−1^), and signal distinguishability (S_s_═|λ_1_‐λ_2_|‐(FWHM_1_ + FWHM_2_), where a positive value indicates no spectral overlap). This provides a standard for optimizing material performance. The Sr_2_P_2_O_7_:Eu^2+^, Eu^3+^ phosphor/PDMS flexible film is combined with a PVA/PAMAA/Gly/NaCl (PPNG) organic hydrogel adhesive layer to fabricate a bio‐inspired skin. This composite is attached to human joints (fingers, wrists, and elbows). Mechanical stress induced by joint bending triggered a ratiometric ML response. For example, when the finger bent, the MLIR $\left(I_{Eu^{2+}}/I_{Eu^{3+}}\right)$ reached 74.0, while it is 10.4 for the wrist and 12.2 for the elbow. The ML color changed from blue to purple accordingly. The strain differences at different body parts are analyzed in real‐time using an optical fiber spectrometer and visual observation. The method overcomes the limitations of traditional ML‐based strain sensing, which is susceptible to environmental interference, by enabling high‐reliability stress visualization through color discrimination. It provides a self‐calibrating solution for wearable health monitoring and human‐machine interaction.

In 2024, Wang et al. proposed a strategy for ratio‐type ML based on a sandwich structure with threshold modulation.^[^ ^86^ ^]^ By innovatively adjusting the initial excitation thresholds of different stress‐sensitive luminescent materials, they achieved dynamic 2D planar luminescence color visualization that depends on applied stress. They selected ML materials with significantly different stress sensitivities (e.g., green‐emitting YGG:Tb^3+^ and red‐emitting LuAGG:Eu^3+^) to construct a multi‐layered flexible composite structure, enabling threshold modulation. The highly sensitive YGG:Tb^3+^ is placed in the middle layer (Layer 2), while the less sensitive LuAGG:Eu^3+^ is positioned in the top layer (Layer 1). The transmittance of the top PDMS composite layer is tuned by adjusting its composition and thickness, allowing selective attenuation of the ML intensity from the middle layer and thereby increasing its initial excitation threshold (Figure 13d).

### 4.3 Human‐Machine Interface

**Figure 14:**
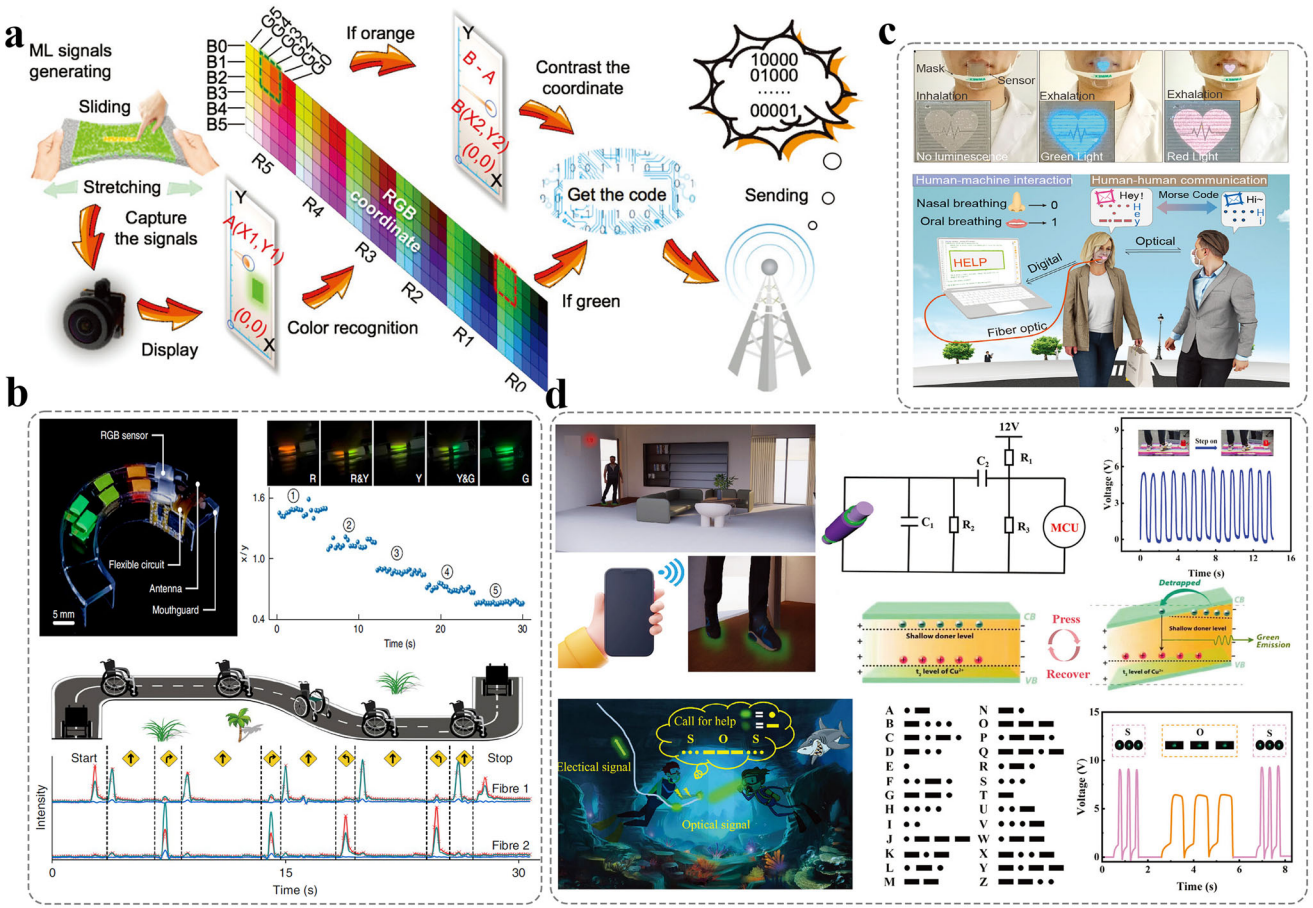
a) Illustrated process of color discrimination and optical trajectory orientation recognition of ML signals, Reproduced with permission.^[^ ^88^ ^]^ Copyright 2022, Wiley‐VCH; b) Photograph of the integrated device comprising an elastomer mouthguard, a bilayer array of multicolor contact pads, and a flexible circuit module, the normalized real‐time chromaticity response in x/y coordinates, derived from five occlusal trajectories comfortable to users. The insets are the corresponding photographs of the mp‐DOF under five force patterns, and the wheelchair navigation using the mouthguard. The motion trajectory of the wheelchair and the corresponding real‐time emission profiles are presented, Reproduced with permission.^[^ ^89^ ^]^ Copyright 2022, Nature; c) Mask integrated with a CUISD for breath‐based interactions and conceptual diagram of the CUISD mask for human‐human interaction and human‐machine interactions, Reproduced with permission.^[^ ^90^ ^]^ Copyright 2024, Wiley‐VCH; d) Schematic representation of an anti‐theft alarm carpet, Reproduced with permission.^[^ ^91^ ^]^ Copyright 2024, Wiley‐VCH.

The 2D stress visualization detection technology transforms abstract mechanical data into intuitive, interactive visual representations. Its technical expansion requires a deep integration of data acquisition and processing, advanced visualization algorithms, and highly responsive interaction design. The goal is to build an efficient visualization analysis environment that can “sense” stress distribution in real time, allow users to “probe” into details, and “reveal” mechanical behavior in an intuitive manner.^[^ ^87^ ^]^ In 2022, Pan et al. developed a self‐powered all‐optical tactile sensing platform (ATSP) based on a heterogeneous ML material system composed of ZnS:Cu and ZnS‐CaZnOS:Mn. This platform enables visual tactile feedback by converting tactile stimuli (such as strain and shear force) into optical signals of different colors, with green emission corresponding to strain and orange emission associated with shear force. As shown in **Figure** **14**a, the system enables users to control a remote‐controlled car (RC Car) and computer games through an integrated tactile glove equipped with ATSP.^[^ ^88^ ^]^ By applying different strains (stretching the joints of the glove) users can generate green light signals corresponding to “switch” commands (binary code “10000”). Alternatively, by performing shear actions (sliding the fingertips) users can produce orange light signals. The motion trajectory of these actions (e.g., left‐right or up‐down directions) is further translated into directional commands (e.g., “01000” for right movement, “00100” for left movement). These optical signals are captured by a camera and transmitted to a microcontroller unit (MCU), which performs color and trajectory recognition. The MCU then sends the command data via a WiFi module to a terminal device. This allows real‐time control of the RC car, including starting/stopping, turning left/right, and accelerating/decelerating, as well as control of computer games, such as block rotation, left/right movement, and acceleration of falling blocks. For the RC car, the control is implemented by adjusting the pulse width modulation (PWM) signal to drive the wheel motors. For the computer game, the control is achieved through a USB interface that communicates with the computer. This system provides a seamless, remote, and eye‐hand coordinated interaction experience.

In the same year, Liu et al. reported a ML driven interactive mouthguard based on mp‐DOF sensors, which consists of a series of elastic waveguides embedded with force‐sensitive phosphor layers that exhibit different colors in response to pressure.^[^ ^89^ ^]^ These sensors are integrated into orthodontic appliances to create an interactive system for controlling occlusion. As shown in Figure 14b, the dental guard utilizes ML materials of ZnS doped with different transition metal ions (e.g., orange emitting ZnS:Cu^2+^/Mn^2+^, green light‐emitting ZnS:Cu^2+^, and blue light‐emitting ZnS:Cu^+^). These materials exhibit distinct spectral characteristics under mechanical stress. By employing the mp‐DOF sensors, the system analyzes the biting position and pattern through colorimetric ratios (CIE tristimulus values). An artificial neural network (ANN) is introduced to classify 14 types of biting trajectories, achieving an accuracy rate of 98%. The device can convert optical signals into control commands via a Bluetooth module, enabling precise control of computers (virtual keyboard input, cursor control), smartphones (emergency calls, application operations), and electric wheelchairs (steering, acceleration). For example, users can navigate a wheelchair along complex paths by using specific biting patterns (e.g., lateral biting to trigger orange light emission), or adjust cursor movement distance based on biting force. The system achieves a response time of less than 0.12 s and an input speed of 22 characters per minute, comparable to the performance of existing brain‐computer interfaces. In addition, the mouthguard is made of biocompatible materials (PDMS, polyurethane), weighs less than 7 g, and features water resistance (light emission intensity retention rate >90% underwater) as well as mechanical stability (signal attenuation <5.5% after 2000 cycles). It offers a low‐invasive and highly adaptable assistive interaction solution for individuals with mobility limitations or neurological disorders. This study advances the application of passive optical sensing technology in smart healthcare and wearable devices through the collaborative innovation of materials, devices, and algorithms.

In 2024, Pan et al. reported a contactless user‐interactive sensing display (CUISD) technology based on humidity‐responsive alternating current electroluminescence (ACEL). By integrating multilayer silver nanofiber electrodes with humidity‐sensitive hydrogel and ZnS:Cu/ZnS:Mn ML particles embedded PDMS composite, the device enables efficient conversion of non‐contact mechanical stimuli into dynamic optical signals.^[^ ^90^ ^]^ Fabricated via electrospinning and microfabrication processes, the patterned electrodes and luminescent layers utilize changes in humidity to modulate hydrogel conductivity, thereby enhancing the electric field across the ACEL layer. This mechanism enables multicolor (orange, green, red) dynamic display with sub‐second response time (0.4 s), while also offering stretchability (stable under 50% strain), large area (up to 10 cm × 10 cm), and high resolution (7569 pixels/3.4 cm^2^). The CUISD system achieves contactless human‐machine interaction through spatial mapping and trajectory tracking of optical signals. As shown in Figure 14c, the CUISD is integrated into a smart mask to distinguish ″0/1″ binary encoding based on respiratory humidity differences (oral vs nasal exhalation light intensity difference >1000 counts). Combined with a Morse code algorithm, it enables visual transmission of emergency messages such as “HELP,” providing a communication method for users with limited mobility.

In the same year, Zhang et al. reported a stretchable self‐powered ML TENG fiber (MLTENGF) based on carbon nanotube fiber.^[^ ^91^ ^]^ Using the synergistic effect between the TENG and ML material, it achieves efficient conversion of mechanical stimuli into dual‐mode electrical and optical signals. The MITENGF features a silicone rubber foam elastic core wrapped with a carbon nanotube fiber (CNTF) electrode and an Ecoflex/ZnS:Cu composite encapsulation layer. Through triboelectrification and carrier recombination luminescence triggered by pressure on the ZnS:Cu material, it maintains stable electrical (VOC up to 28 V) and optical signal outputs even under 200% tensile strain, with a non‐contact sensing range of 35 cm. As illustrated in Figure 14d, the MITENGF is woven into a pressure‐sensing carpet to create an intelligent interactive interface. Electrical signals triggered by foot movements enable human gait recognition (walking, running, jumping). Additionally, a 3 × 3 pixel array is constructed to provide real‐time visual feedback of spatial pressure distribution.

### 4.4 Near‐Distance ML Imaging Sensor

**Figure 15:**
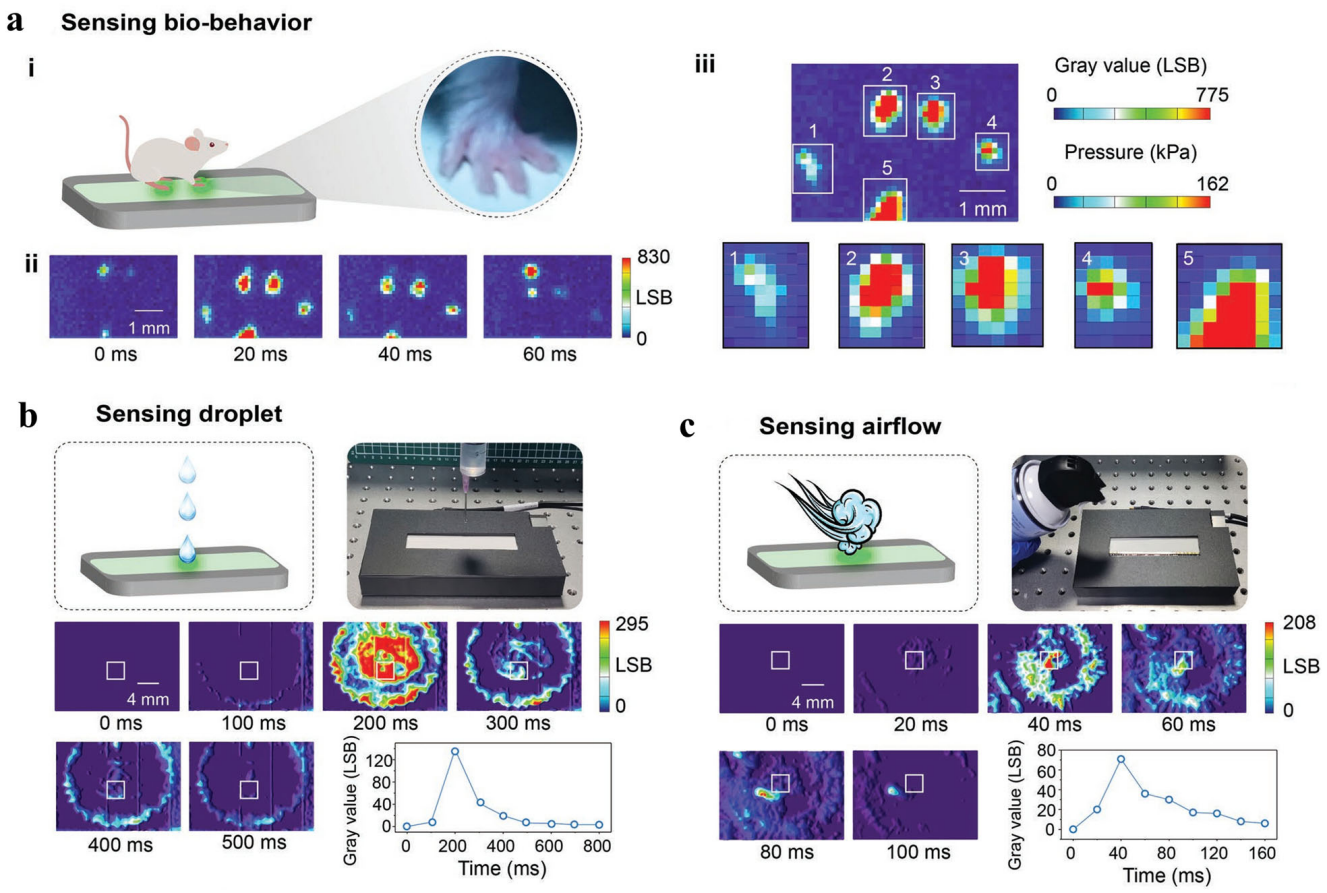
Demo applications of the near‐distance ML imaging sensor. a) Sensing bio‐behavior. Schematic diagram of the sensor being clawed by a mouse (i). Pseudo‐color images showing gray value distribution. The four images are taken at different time. Frame rate is 50 fps (ii). Pressure distribution on pixels derived from gray value distribution. At the bottom are enlarged images of five selected areas (iii); b) Sensing droplet; c) Sensing airflow. The figures include a schematic diagram, a photographic image of the sensor, pseudo‐color images at different time, and the average gray value of the selected area as a function of time. The frame rates in (b,c) were 10 and 50 fps, respectively, Reproduced with permission.^[^ ^92^ ^]^ Copyright 2022, Wiley‐VCH.

In close‐range imaging, it enables real‐time, high‐resolution stress visualization, allowing rapid analysis of in‐plane stress distribution through 2D stress mapping technology. In 2022, Zhuang et al. proposed an innovative near‐field ML imaging approach by depositing the ML film directly onto the surface of a CMOS image sensor, reducing the distance between them to nearly zero.^[^ ^92^ ^]^ This configuration significantly enhances photon collection efficiency. Compared to conventional remote imaging methods, the photon harvesting rate could be up to 80%, resulting in a two order of magnitude improvement in sensor sensitivity. Furthermore, they enhanced the mechano‐photonic conversion efficiency of the ML film by incorporating Al_2_O_3_ nanoparticles into a ZnS:Cu@PDMS composite. They found that with 10 wt.% of 100 nm nanoparticles, the ML intensity will significantly increase. This enhancement is attributed to a moderate rise in the elastic modulus of the film from 1.62 to 1.83 MPa, which promotes greater deformation at the interface between ZnS:Cu particles and the PDMS elastomer under dynamic mechanical stimulation. Through systematic investigation of various oxide nanoparticles (Al_2_O_3_, ZrO_2_, SiO_2_, In_2_O_3_, MgO, TiO_2_) and their effects on ML performance, the team established a clear correlation between elastic modulus and ML intensity. The aforementioned near‐field ML imaging sensor achieves three breakthrough performance metrics: a detection sensitivity at the kPa level (capable of detecting pressures as low as 20 kPa), a spatial resolution of 254 dpi (pixel size: 100 µm × 100 µm), and a rapid response capability of 300 fps (with an interval of 3.3 ms). These performance metrics are validated through carefully designed experiments. As shown in **Figure** **15**, an impact test using a 518 mg ball dropped from a height of 8 cm demonstrated that the sensor captures the maximum deformation within 3.3 ms and fully records the elastic recovery dynamics over a 30 ms decay process. Furthermore, the near‐field ML imaging sensor is explored for real‐time imaging in biomechanical monitoring, water droplet impact, and high‐speed airflow. In sensing bio‐behavior applications, it successfully captured the dynamic stress distribution generated by a mouse paw during grasping. Grayscale analysis enabled quantification of pressure distribution across Area 5 (reaching up to 162 kPa) and the total applied force (27.1 mN). This high‐sensitivity detection offers a tool for animal behavior studies and tactile feedback in bioinspired robotics. In sensing droplet research, the sensor achieved real‐time imaging of water droplet impact (a 16.7 mg droplet released from 20 cm height) and high‐speed airflow (0.5 MPa).

## 5 3D Stress Visualization Detection

**Figure 16:**
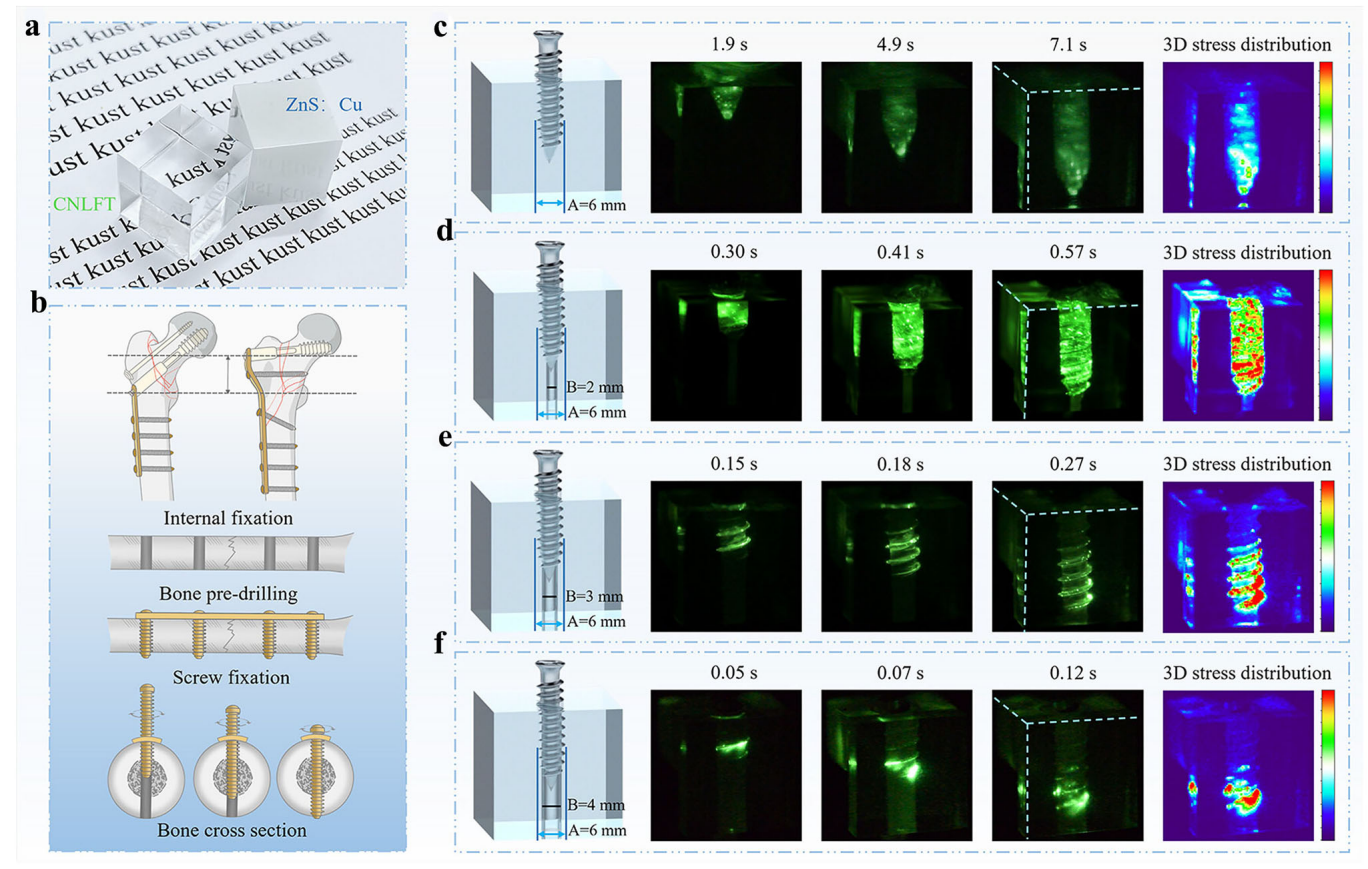
a) 3D printed cubes of NaLuF_4_:0.15Tb^3+^ nanocrystals and the commercial ZnS: Cu micron crystals; b) Schematic diagram of the internal fixation procedure via driving screws into the steel plate for a broken bone; c–f) Dynamic 3D ML imaging for the screw drilled into the cube with predrilled apertures, Reproduced with permission.^[^ ^22^ ^]^ American Chemical Society.

The 3D stress visualization detection technology reconstructs the entire stress field within a 3D space to achieve a comprehensive analysis of the multi‐axis stress distribution within complex structures. The key lies in integrating luminescent materials with advanced imaging technology, and by capturing the fluorescence intensity distribution of the materials in 3D space, a 3D stress distribution model can be constructed. At present, the research on visual detection of 3D stress distribution is rarely explored. As demonstrated by Xu et al., synergistic regulation of the trap structure via Cs⁺ ion doping and X‐ray irradiation successfully introduced trap energy levels with depths of 0.88 and 1.02 eV in NaLuF_4_:0.15Tb^3+^ nanocrystals (average particle size of ≈25 nm).^[^ ^22^ ^]^ Using these materials, a nanocrystal ink is developed and used to fabricate transparent cubes via 3D printing. As shown in **Figure** **16**, the linear relationship between ML intensity and mechanical stress enables real‐time visualization of internal stress distribution in non‐planar structures, such as during orthopedic screw implantation. The system clearly reveals stress concentration zones (e.g., at the screw tip) and stress distribution gradients under without and with predrilled (2–4 mm) conditions, achieving submillimeter spatial resolution and millisecond temporal resolution. This approach provides 3D stress visualization capabilities for studying mechanical behavior in complex curved structures.

## 6 Summary and Outlook

Utilizing their unique stress‐to‐light conversion capability, ML materials have enabled stress sensing to evolve from traditional single‐point detection to 3D stress field visualization, demonstrating distinct advantages in fields such as materials science, biomedical engineering, and structural health monitoring. This review systematically summarizes recent advances in multi‐dimensional stress sensing of ML material in 0D, 1D, 2D, 3D applications. From a material performance perspective, strategies such as activator ion selection, bandgap engineering, and defect regulation have significantly enhanced stress‐to‐light conversion efficiency. In terms of sensor design, layered structural configurations have remarkably improved both performance and environmental adaptability of ML sensing systems. Spatially, resolution continues to improve through controlled particle size optimization and advances in imaging technology. Nevertheless, research on multi‐dimensional ML still faces several critical challenges, including: 1) On the material performance front, the simultaneous optimization of emission intensity, spatial resolution, and environmental stability remains challenging due to significant trade‐off relationships among these properties. 2) In terms of device integration, achieving efficient and stable compatibility between ML sensing units and existing engineering monitoring systems needs to be addressed. 3) Regarding data processing, the development of high‐performance algorithms is essential to accurately extract quantitative mechanical information from complex spatiotemporal dynamic optical signals. Addressing these challenges requires a deep interdisciplinary integration of materials science, optical engineering, mechanics, and information science.

Looking forward, stress visualization technology is advancing toward higher‐dimensional capabilities. For example, machine learning is widely employed in material design and selection. Its introduction has transformed materials research and development from a traditional “trial‐and‐error” approach into an efficient “rational design” paradigm. By establishing quantifiable “composition‐structure‐process‐property” relationships through data‐driven methods, this shift enables a transition from reliance on experiential intuition to science‐based prediction. Thus, machine learning can be employed for material design and screening, while the introduction of temporal or thermal dimensions to 3D systems enables the construction of higher‐dimensional frameworks. Achieving the above technology requires breakthroughs in the following key aspects:

### 6.1 Advanced ML Material Design and Preparation

The development of next‐generation ML materials demands a shift from empirical approaches to intelligent design. By integrating machine learning with materials science, we can systematically develop material systems exhibiting high sensitivity, fast response, excellent environmental stability, and mechanical reversibility. Machine learning algorithms can analyze vast datasets of material compositions and processing parameters to identify optimal strategies such as rare earth doping combinations, composite structural designs, and surface modification techniques. Through predictive modeling, ML can forecast how specific doping elements will affect trap energy levels and charge carrier dynamics, while generative design algorithms can propose novel composite architectures that maximize stress transfer efficiency while minimizing mechanical hysteresis. Furthermore, active learning frameworks can guide the experimental optimization of synthesis parameters to simultaneously enhance optical performance and fatigue resistance, creating a closed‐loop design system that continuously improves material performance based on real‐time feedback from characterization data.

### 6.2 High‐Precision Dynamic Signal Acquisition System

This system establishes an advanced experimental platform for capturing transient ML phenomena by integrating highly sensitive photodetectors with multi‐field coupling loading devices. Through the incorporation of machine learning algorithms, the system can deliver breakthroughs in four core aspects: Deep learning‐based intelligent noise reduction enhances weak signals through trained ML characteristic models. Adaptive synchronization control employing predictive algorithms achieves millisecond‐level coordination among stress loading, optical imaging, and signal acquisition; automated anomaly detection during dynamic testing enables real‐time identification of abnormal stress‐optical response patterns; and convolutional neural networks facilitate real‐time reconstruction of raw optical data into high spatiotemporal resolution stress field mappings. This intelligent platform not only significantly improves the accuracy of dynamic stress field visualization but also advances cutting‐edge stress sensing applications through predictive analytics.

### 6.3 Intelligent Data Processing and Dynamic Reconstruction

This system employs convolutional neural networks and spatiotemporal sequence prediction models to perform real‐time decoupling, denoising, and feature extraction from massive luminescence data. By establishing the light‐stress‐temporal coupling model, it will accomplished visual reconstruction and quantitative inversion of dynamic stress field evolution. Further integration of deep learning architectures enables the development of temporal prediction models for stress evolution, achieving the critical transition from stress state monitoring to early damage warning while forming a comprehensive closed‐loop diagnostic framework. Through continuous learning mechanisms, the system progressively refines model accuracy, delivering ever‐evolving analytical capabilities for structural health monitoring applications.

In conclusion, future investigations can explore 5D and even higher‐dimensional stress detection systems, by integrating additional physical dimensions such as thermal and electric fields with existing spatiotemporal stress data. Such evolution in sensing capabilities would create novel pathways for implementing intelligent full‐lifecycle monitoring systems in critical engineering structures and equipment, where simultaneous tracking of multiple physical parameters could substantially enhance the precision and reliability of structural integrity assessment.

## Conflict of Interest

The authors declare no conflict of interest.
